# A systematic review of social network sentiment analysis with comparative study of ensemble-based techniques

**DOI:** 10.1007/s10462-023-10472-w

**Published:** 2023-04-12

**Authors:** Dimple Tiwari, Bharti Nagpal, Bhoopesh Singh Bhati, Ashutosh Mishra, Manoj Kumar

**Affiliations:** 1 Ambedkar Institute of Advanced Communication Technologies and Research (GGSIPU), Delhi, India; 2 NSUT East Campus (Formerly Ambedkar Institute of Advanced Communication Technologies and Research), Delhi, India; 3 Indian Institute of Information Technology, Sonepat, Haryana, India; 4 School of Integrated Technology, Yonsei University, Seoul, South Korea; 5 Faculty of Engineering and Information Sciences, University of Wollongong in Dubai, Dubai Knowledge Park, Dubai, UAE; 6 Department of Electronics & Communication Engineering, Graphic Era Deemed to be University, Dehradun, India; 7 MEU Research Unit, Faculty of Information Technology, Middle East University, Amman 11831, Jordan

**Keywords:** Natural language processing, Machine learning, Ensemble techniques, Bagging, Boosting, Social-networks

## Abstract

Sentiment Analysis (SA) of text reviews is an emerging concern in Natural Language Processing (NLP). It is a broadly active method for analyzing and extracting opinions from text using individual or ensemble learning techniques. This field has unquestionable potential in the digital world and social media platforms. Therefore, we present a systematic survey that organizes and describes the current scenario of the SA and provides a structured overview of proposed approaches from traditional to advance. This work also discusses the SA-related challenges, feature engineering techniques, benchmark datasets, popular publication platforms, and best algorithms to advance the automatic SA. Furthermore, a comparative study has been conducted to assess the performance of bagging and boosting-based ensemble techniques for social network SA. Bagging and Boosting are two major approaches of ensemble learning that contain various ensemble algorithms to classify sentiment polarity. Recent studies recommend that ensemble learning techniques have the potential of applicability for sentiment classification. This analytical study examines the bagging and boosting-based ensemble techniques on four benchmark datasets to provide extensive knowledge regarding ensemble techniques for SA. The efficiency and accuracy of these techniques have been measured in terms of TPR, FPR, Weighted F-Score, Weighted Precision, Weighted Recall, Accuracy, ROC-AUC curve, and Run-Time. Moreover, comparative results reveal that bagging-based ensemble techniques outperformed boosting-based techniques for text classification. This extensive review aims to present benchmark information regarding social network SA that will be helpful for future research in this field.

## Introduction

With the incremental growth of information technology and social platforms, user-generated information can easily be posted online, and this information contains people's sentiments and emotions toward a particular issue. While government, companies, and individuals are interested in retrieving the sentiments behind that reviews. Miserably, with the massive amount of data, it is challenging to polarize these comments and reviews. Where human experts are overpriced for labeling these reviews manually. Accordingly, SA is gaining a lot of popularity in research topics (Chen and Yang 2011). It is a broadly active method for analyzing and extracting opinions from text using individual or ensemble learning techniques. This field has unquestionable potential in the digital world and social media platforms. The vast content generated on the web is unstructured, which can be processed by the SA and converted into meaningful information. SA is the subset of NLP that combines computational linguistics, a rule-based approach, and machine learning for extracting the public's opinion from content provided on social platforms, including text, images, and videos. According to the requirement of a particular application, the problem of sentiment classification is primarily handled at aspect, sentence, and document levels. Aspect-based SA is known as the feature-level SA in which multiple features are extracted from the text reviews. Aspect-based SA provides a deep study of reviews and extracts the context of reviewers for a particular domain (Thet et al. 2010; García-Pablos et al. 2018). The aspect-level approach mainly depends on the syntactic features of the text reviews (Che et al. 2015). Sentence-based SA approach works on finding the polarity for a particular sentence. Here, the various words are linked together to form a sentence and extract the polarity from that sentence N-Grams technique is used, which separates the words into pair of one, two, or maybe three. Sometimes N-Gram technique is failed to find the relationship between these words. Therefore, dependency tree and typed dependency have been introduced to address the word separation problem in text classification (Meena and Prabhakar 2007). In the sentence-level classification, each sentence is considered a separate unit and assumes that every sentence produces only one opinion: positive, negative, or neutral (Jagtap and Pawar 2013). Each document is considered a single unit in the document-based approach, and a single opinion is assigned for the whole document. The Bag-of-words approach is very popular and provides more accuracy in handling complexity in document-level SA (Bhatia et al. 2015). Most sentence-level applications try to achieve good accuracy in the whole document (Zhang et al. 2009). SA and opinion mining are two popular fields that help to calculate opinioned information from online social platforms. These are commonly reciprocal to present a similar meaning. However, some researchers are used them for handling slightly different problems. SA is used to detect the sentiment from reviews as neutral, negative, or positive, and opinion mining is used to analyze a text's subjectivity (Tsytsarau and Palpanas 2012). Previous research employed machine learning and heuristic-based methods very frequently. Heuristic-based methods mainly depend on semantic features and linguistic characters, whereas machine learning-based algorithms are classified into unsupervised, supervised, and ensemble learning.

**Fig. 1:**
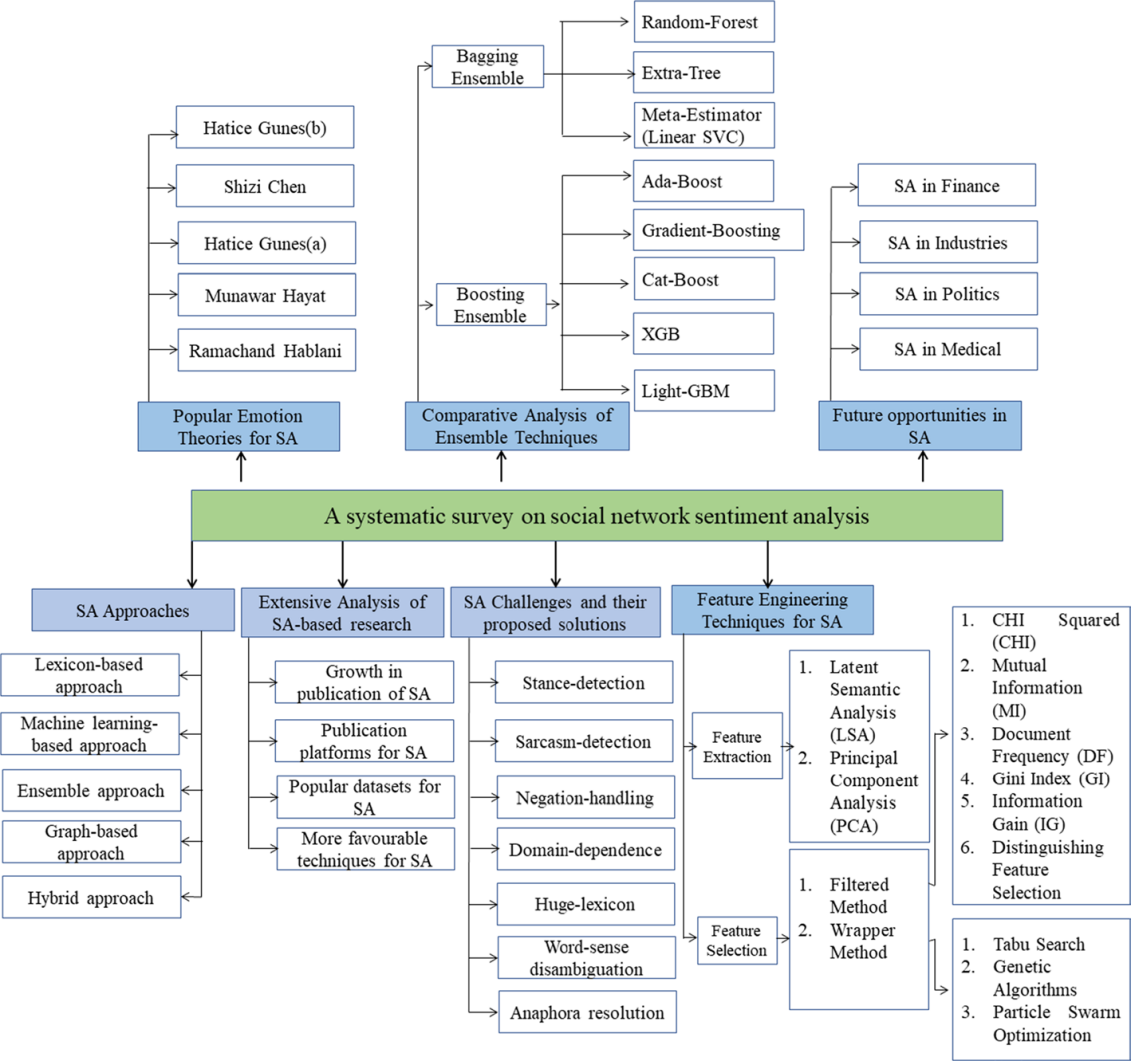
Taxonomy of social network SA

Several articles have been published related to SA using different techniques, which generates a need for a deep study to summarize the trends and aspects related to SA. One comparative study and one detailed survey were also presented a few years back by Xia et al. (2011) and Giachanou and Crestani (2016) in 2011 and 2016, respectively. Xia et al. (2011) provided a comparative study of ensemble-based techniques for SA but did not cover the advanced ensemble approach of this field. Giachanou and Crestani (2016) presented an in-depth survey related to Twitter SA and summarized the previously proposed approaches of SA in Twitter. However, this survey did not implement any latest techniques for comparative discussion and did not explore the latest updates in this field. Here, we provide a detailed SA survey and present all the recent facts and trends related to this field. This study investigated the research work from 1996 until 2022 utilizing online repositories and tried to cover all the essential aspects related to SA, which will provide deeper information to upcoming researchers in a single manuscript. Extensive experiments have also been conducted on different domains to provide the best ensemble approach for the sentiment classification task—this analytical study was mainly conducted for sentence-level SA using ensemble machine-learning techniques. Furthermore, experimented ensembles are categorized into two major categories; bagging and boosting. Accordingly, eight ensemble learners were implemented, where five belonged to boosting approach and three from the bagging approach. Figure 1 presents the summarized taxonomy of our social network SA survey.

Multiple learners learn together in an ensemble approach to get more accurate and efficient results than individual learners. Ensemble methods have been used in NLP applications and are proven better than a single method (Zhang et al. 2009). The Convolutional Neural Network (CNN) and Long Short-Term Memory (LSTM) models with averaging method generate better results than individual ones (Minaee et al. 2019). Although governments, businesses, and individuals are always interested in calculating the polarity and sentiment from the reviews, no consistent conclusion is available to prove which methodology is best for this process. Therefore to find conclusive results, this study compares eight ensemble techniques on four popular datasets to investigate the performance of ensemble models for SA. The main objective of this study is to explore the latest research on sentiment classification with a comparative analysis of ensemble-based techniques. Therefore, we explained five research queries.

- *RQ*1 What are the different approaches, publishing platforms, and benchmark datasets used by researchers for the SA.
  To discover the most popular approach and dataset used in the field of SA. This would be helpful for the researchers to understand the current scenario related to this area.
- *RQ*2 What are the major challenges facing the researchers during sentiment calculation from text reviews.
  Discuss the challenges in the field of NLP with their proposed solutions.
- *RQ*3 What are the distinct feature engineering techniques for selecting the essential features from text reviews.
  To explain the various feature engineering techniques for dimensionality reduction of text datasets. Thus, many critical research papers have been collected from different publishing sites to map popular feature engineering techniques for text datasets.
- *RQ*4 What are the researchers' emotion theories to detect the emotions from the social content, including text, images, and videos.
  To identify the common emotions that are present in prestigious theorist emotion sets. It would provide the best emotion set to future researchers for opinion extraction from the social content, including text, images, and videos.
- *RQ*5 Which is the best ensemble technique for sentiment classification and future opportunities of SA.
  To discover the best ensemble technique this provides the highest results in terms of all standard measures. Hence, various experiments were conducted on different domains to select the best technique of text classification. It would be helpful for SA-related applications. Future opportunities related to SA have been discussed.

The further sections of this study are categorized as follows: Sect. 2 presents the extensive literature survey related to the SA. Section 3 elaborates on the all-important aspects of SA. Section 4 describes the methodology used for the comparative study. Section 5 presents the comparative results and analysis. Section 6 discussed the future opportunities of SA. Finally, Sect. 7 generates the study's conclusion and addresses some needful issues for future research.

## Literature survey

**Fig. 2:**
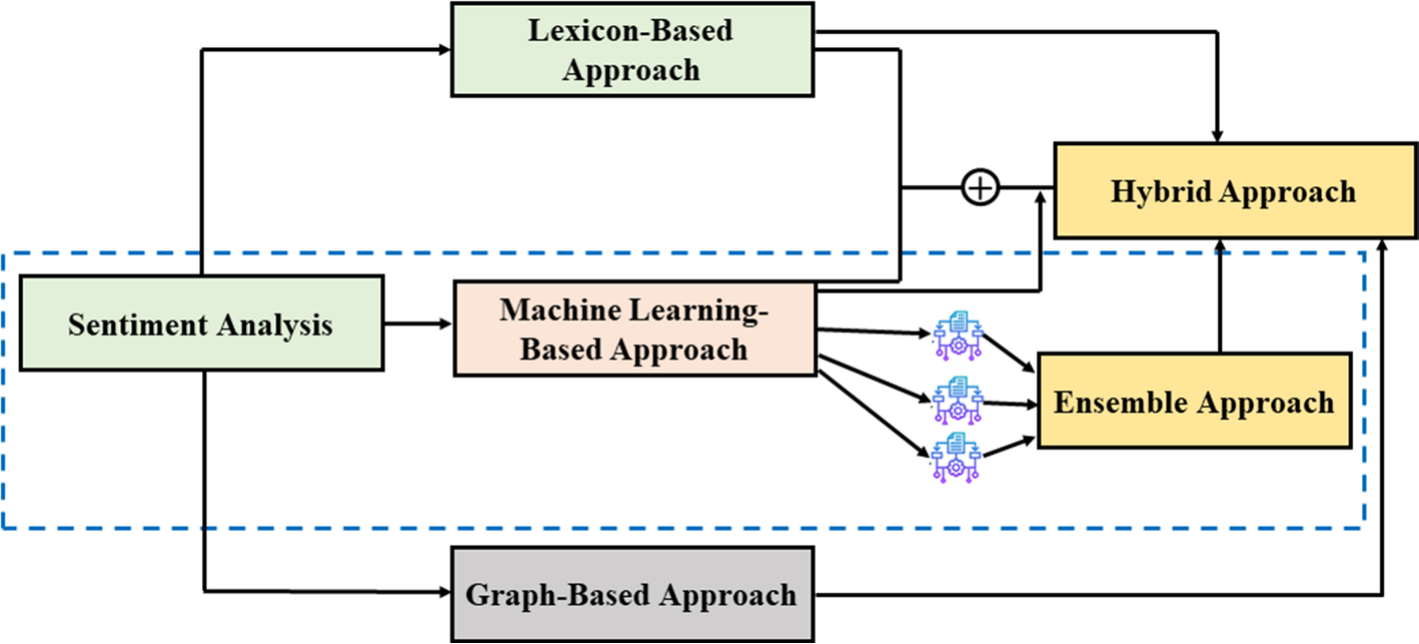
Classification of proposed SA approaches

SA is extensively used to extract people's opinions, emotions, and sentiments toward a particular brand, business, place, or product. Various techniques and approaches are also introduced to classify the sentiments as the demand for SA increases. After analyzing the vast literature on sentiment classification, we have concluded that SA can use five significant approaches. Figure 2 presents the classification of all the major approaches used by researchers for sentiment classification.

First, the lexicon-based approach uses a manually or automatically-generated list of various positive, negative or neutral polarity terms for sentiment classification. The lexicon approach computes the semantic orientation of phrases and words in sentences and documents to reveal the sentiments. Usually, the lexicon-based approach uses adjectives to indicate the semantic adjustments (Taboada et al. 2011). Second, the machine learning approach is a widely adopted technique for SA. Most researchers preferred a machine learning-based approach for sentiment classification due to their fast execution and reliable results. Machine learning provides various single learners, namely Naïve Bayes (NB), K-Neighbors (KN), Linear Regression (LR), Support Vector Machine (SVM), and so forth. Third, the graph-based approach selects the nodes and vertices based on the feature (reviews and tweets) available in input materials. Various graph-based models such as Enterprise Graphs, Hyper-graph, Hashtag Graphs, N-Gram Graph, and Co-Occurrence Graph are available for effective SA process (Krishnakumari and Akshaya 2019). Fourth, the ensemble approach combines multiple weak learners to form a powerful learner. Various ensemble learners, namely Random-Forest, Extra-Tree, Meta-Estimator, Ada-Boost, Gradient-Boosting, Light-GBM, Cat-Boost, and Extreme Gradient-Boost, are available to make the sentiment process more effective than the lexicon approach and single machine learning approach. Fifth, the most potent Hybrid approach that enhances the capability of sentiment classification model with the integration of machine learning and lexicon-based approach or with the combination of multiple machine learning algorithms. A hybrid approach is a novel idea that the researchers present to build a more prosperous and robust model for solving a particular problem. The researcher performs various experiments with discriminant techniques on specific data and tries to create a more effective model than a single and ensemble model. For example, linguistic dictionary and SVM were combined to build a hybrid model for political tweets sentiment classification that acquired 93% accuracy for sentiment classification, which is significant enough and beneficial for politicians to make strategies for future elections (Nandi and Agrawal 2016). Here, we categorized all the previous research into two parts: Sentiment Analysis (SA)—which studies the subjective information in the text, and Sentiment Classification (SC)—which identifies the opinions from the text and assigns a particular label to them.

### Lexicon-based approach

Phrases and opinions implement lexicon-based approaches without prior knowledge of labels. Here, collective phrases are treated as an opinion lexicon along with negative and positive words. Opinion lexicons determine the orientation of the terms available in the text dataset. The lexicon-based approach is categorized into two parts; the Dictionary-Based approach- judges the sentiment based on phrases available in lexicons, and the Corpus-Based approach—extracts the context present in the text. Table 1 reports the list of lexicon-based research from 2011 to 2022.

**Table 1:** Lexicon-based approach for SA from 2011 to 2022

Author	Year	SA	SC	Dataset	Approach	Cited
Taboada et al. (2011)	2011	✓		Original collection 400 reviews (Taboada and Grieve 2004), New collect 400 texts from epinions.com, 1900 polarity text (Pang and Lee 2004)	Dictionary-based	2796
Sharmaa et al. (2012)	2012	✓		Movie and hotel reviews	Dictionary-based	37
Abdul-Mageed et al. (2012)	2012	✓		Modern STANDARD Arabic (MSA)	Corpus-based	188
Palanisamy et al. (2013)	2013	✓		SemEval-2013 Task (Twitter)	Dictionary-based	81
Kundi et al. (2014)	2014		✓	Twitter	Dictionary-based	59
Kaushik et al. (2014)	2014	✓		Twitter	Dictionary-based	53
Abdulla et al. (2014)	2014	✓		Twitter Corpus, Yahoo!-Maktoob Corpus	Dictionary-based	82
Bhoir et al. (2015)	2015			Movie-reviews-dataset	Corpus-based	20
Moreno-Ortiz et al. (2015)	2015	✓	✓	General language polarity database	Corpus-based	32
Rajput et al. (2016)	2016	✓		Student feedback	Dictionary-based	35
Akter et al. (2016)	2016	✓		Facebook	Dictionary-based	29
Ray et al. (2017)	2017	✓		Twitter	Dictionary-based	28
Aung et al. (2017)	2017		✓	Manually created lexical for students’ feedback	Dictionary-based	40
Alshutayri et al. (2017)	2017	✓		Twitter	Corpus-based	22
Khoo et al. (2018)	2018		✓	Amazon product reviews	Dictionary-based	60
Dey et al. (2018)	2018		✓	Movie reviews	Dictionary-based	31
Tiwari et al. (2019)	2019	✓		YouTube	Dictionary-based	1
Yerpude et al. (2019)	2019			Amazon customer reviews	Dictionary-based	1
Mowlaei et al. (2020)	2020		✓	Customer reviews (Bing Liu’s)	Dictionary-based	8
Atteveldt et al. (2021)	2021	✓		Newsgroups	Dictionary-based	86
Sallam et al. (2022)	2022	✓		Book reviews	Corpus-based	3

### Machine learning-based approach

Machine learning is the most promising approach for SA. Usually, machine learning-based SA provides a high accuracy score than the lexicon-based approach. It offers various feature engineering techniques that extract the critical features from the dataset and improve the efficiency of SA. Different supervised and unsupervised algorithms are available for sentiment classification. The supervised approach works on labeled datasets and uses a mapping function to map the input labels with output labels. In contrast, unsupervised learning learns the pattern from unlabeled data using clusters. Table 2 presents a few popular pieces of research related to machine learning-based SA from 2011 to 2022.

**Table 2:** Machine learning-based approach for SA from 2011 to 2022

Author	Year	SA	SC	Dataset	Approach	Cited
Saleh et al. (2011)	2011	✓		Amazon reviews	SVM	214
Tan et al. (2011)	2011	✓		Twitter	SVM	483
Wang et al. (2012)	2012	✓		Movie reviews	NB, SVM	1130
Habernal et al. (2013)	2013	✓		Facebook corpus, movie reviews, product reviews	Maximum Entropy (ME), SVM	70
Anjaria et al. (2014)	2014	✓		Twitter	SVM, NB, ME, Artificial neural network (ANN)	46
Patil et al. (2014)	2014	✓		Product reviews	SVM	37
Le et al. (2015)	2015	✓		Twitter	NB, SVM	39
Wawre et al. (2016)	2015		✓	Movie reviews	NB, SVM	56
Dey et al. (2016)	2016	✓		Hotel reviews, movie reviews	NB, K-Nearest Neighbors (KNN)	123
Elmurngi et al. (2017)	2017	✓		Movie reviews V2.0 &V1.0	NB, SVM, J48, KNN-1BK, KStar (K*)	16
Singh et al. (2017)	2017	✓		Amazon reviews, IMDB reviews	NB, J48, BFTree, OneR	56
Hasan et al. (2018)	2018	✓		Twitter	SVM, NB	115
Shi et al. (2011)	2019	✓		Hotel reviews	SVM	78
Jagdale et al. (2019)	2019	✓		Amazon reviews	NB, SVM	23
Kumar et al. (2020)	2020	✓		Facebook reviews	ME, SVM	5
Usman et al. (2021)	2021		✓	Forensic reviews	Decision Tree (DT)	58
AIBadani et al. (2022)	2022		✓	Tweets, IMDB Reviews, GOP debate	SVM	12

### Graph-based approach

A graph-based approach connects interrelated words in text reviews to calculate the sentiment and opinion of people where vertices and nodes conform to features available in reviews. Various graph-based methods and algorithms have been applied in the last decades to solve the problem of SA. Table 3 visualizes the multiple pieces of research that have been done in the area of SA using graph-based methods.

**Table 3:** A graph-based approach for SA from 2011 to 2022

Author	Year	SA	SC	Dataset	Approach	Cited
Aisopos et al. (2011)	2011	✓		Twitter	N-Graph	60
Ponomara et al. (2012)	2012		✓	Product reviews	OPTIM, RANK algorithm	37
Rajagopal et al. (2013)	2013		✓	Manual reviews	POS-based bigram algorithm	82
Montejo-Ráez et al. (2014)	2014		✓	Twitter	Random walk algorithm	157
Castillo et al. (2015)	2015	✓		SMS	Co-occurrence graph	15
Violos et al. (2016)	2016	✓		Twitter	Word graph method	11
Chet et al. (2017)	2017		✓	Book, DVD reviews	Hyper-graph	4
Westgate et al. (2018)	2018		✓	Self-generated	Word-graph	2
Liu et al. (2019)	2019	✓		Stock reviews	Enterprise knowledge graph	9
Bordoloi et al. (2020)	2020	✓		Customer reviews	Co-occurrence graph	0
Xia et al. (2021)	2021	✓		Customer reviews	Relational graph	11
Liang et al. (2022)	2022	✓		Tweets and reviews	Graph convolutional network	80

### Ensemble approach

Ensemble learning is a process of combining several learners strategically to form an intelligent model. It improves the classification problem by reducing poor and unfortunate selection. It has the capability and knowledge of various learners, which increases the accuracy of a classification and decreases errors in prediction. Decision-based on diverse learning makes ensemble learning more accurate and trustworthy than single learning. Table 4 presents the research work done in SA using an ensemble approach from 2011 to 2022.

**Table 4:** Ensemble-based approach for SA from 2011 to 2022

Author	Year	SA	SC	Dataset	Approach	Cited
Xia et al. (2011)	2011		✓	Reviews (movie, book, DVD, elec, kitchen)	Fixed-combination, weighted-combination, meta-estimator	589
Su et al. (2012)	2012		✓	Product Reviews	Stacking	41
Sharmab et al. (2013)	2013	✓		Movie & hotel reviews	Boosting	8
Fersini et al. (2014)	2014		✓	Gold standard dataset (Twitter)	Bayesian averaging	149
Wang et al. (2014)	2014		✓	Product Reviews	Bagging, boosting, random sub-space	284
Chalothom et al. (2015)	2015	✓		Twitter	Majority voting	36
Perikos et al. (2016)	2016		✓	News, Twitter	Voting	105
Lochter et al. (2016)	2016		✓	Product Reviews	Majority voting	38
Onana et al. (2016)	2016		✓	Laptop reviews	Weighted voting	122
Onanb et al. (2016)	2016		✓	ACM collection reviews	Bagging, boosting, voting	185
Heredia et al. (2017)	2017		✓	Doctors, hotels, and restaurants reviews	Select-Boost, select-bagging, random-forest	9
Ramesha et al. (2017)	2017	✓		Medicine Reviews	Bagging, boosting	0
Akhtar et al. (2017)	2017	✓		SemEval-2014 (Twitter)	Majority, weighted voting	95
Saleena et al. (2018)	2018	✓		Twitter	Majority voting	49
Bari et al. (2018)	2018	✓		Twitter, IMDB reviews, Yelp, Amazon reviews	Multiveriate-regression, majority voting	5
Kim et al. (2019)	2019	✓		Food Reviews	Boosting	2
Khan et al. (2019)	2019	✓		Reviews (movie, book, DVD, elec, kitchen)	Majority voting	3
Khalid et al. (2020)	2020		✓	Mobile reviews	Voting	1
Nazeer et al. (2021)	2020	✓		Twitter	Majority voting	0
Bibi et al. (2022)	2021	✓		Twitter	Ensemble hierarchal clustering	19
Rahman et al. (2022)	2022		✓	Twitter	Stacking ensemble	17

### Hybrid approach

The hybrid approach utilizes the capability of various approaches such as rule-based, lexicon-based, machine learning, or deep learning-based. It enhances the efficiency of the SA model with optimum results. It is an idea that generates in a researcher's mind to develop the best approach for a particular task. Hybrid learning is categorized into semi-supervised learning, multi-instance learning, and self-supervised learning. A semi-supervised learning trains with very few predefined labels and classifies a large amount of unlabeled text. Multi-instance learning does not contain individual labels; instead, it receives labeled bags, and each bag has various instances, which explicitly treats the problems with incomplete knowledge of training examples. Self-supervised learning generates labels by itself and utilizes supervised algorithms to solve unsupervised problems. Hybrid models show significantly more improvement in classification than other methods. Table 5 presents work related to hybrid SA.

**Table 5:** Hybrid-based approach for SA from 2011 to 2022

Author	Year	SA	SC	Dataset	Approach	Cited
Roman et al. (2011)	2011		✓	Reviews	Machine learning with a rule-based system	38
Sohn et al. (2012)	2012		✓	Suicide notes	Machine learning with a rule-based system	28
Govindarajana et al. (2013)	2013		✓	Movie reviews	Coupling Classification (NB & genetic algorithm)	43
Revathy et al. (2013)	2013		✓	Twitter	Combination of rule-based classification, sense-based classification, and entity-level analysis	10
Govindarajanb et al. (2014)	2014		✓	Restaurant reviews	Coupling classification (NB, SVM, genetic algorithm)	27
Al-Twairesh et al. (2014)	2014	✓		SemEval-2014 (Twitter)	Rule-based, Lexicon-based, and Machine learning	40
Bhaskar et al. (2015)	2015		✓	SemEval-2007 (Twitter)	Rule-based with machine learning	52
Asghar et al. (2016)	2016		✓	Online health reviews	Bootstrapping	44
Nandi et al. (2016)	2016	✓		Twitter	Combination of lexicon and machine learning approach	6
Mondal et al. (2016)	2016		✓	Medical reviews	Combination of Lexicon with a machine learning approach	11
Keith et al. (2017)	2017	✓		Paper reviews	Combination of Lexicon with a machine learning approach	4
Tripathy et al. (2017)	2017		✓	IMDB reviews	Machine learning with deep learning	41
Amrani et al. (2018)	2018	✓		Amazon reviews	Machine learning	45
Zainuddin et al. (2018)	2018		✓	Twitter	Machine learning	61
Srivasatva et al. (2019)	2019	✓		Twitter	Machine learning	9
Joshi et al. (2019)	2019	✓		Twitter	Combination of Lexicon with a machine learning approach	3
Elshakankery et al. (E^2019^)	2019	✓		Twitter	Combination of Lexicon with a machine learning approach	17
Chaithra et al. (2019)	2019	✓		Mobile reviews	Combination of Lexicon with a machine learning approach	4
Banlahbib et al. (2020)	2020	✓		Product Reviews	Machine learning	7
Jing et al. (2021)	2021		✓	Stock Reviews	Combine deep learning models	62
Tiwari et al. (2022)	2022		✓	Covid-19 and Indian Farmer Protest associated Tweets	Hybrid transformer approach	1

### Extensive literature analysis

This section presents a deep analysis of literary work that has been done in the field of SA. Various graphs and tables have been used to discuss algorithms, datasets, approaches, and the most popular platforms related to SA. It has been employed in numerous real-life applications. Therefore researchers take more insight into it. Hence, this section focuses on various essential points that are required for further research in this area.

#### Growth in publications of SA

**Fig. 3:**
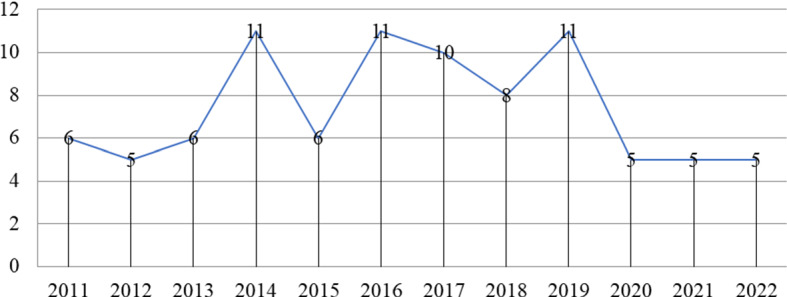
Number of publications per year related to SA set (N = 92)

This section shows the growth in the number of publications of SA. As shown in Fig. 3, the number of publications related to SA was very few in starting years (2010, 2011, and 2013). As the demand for social platforms has increased, publications associated with SA have also emerged since 2014. In 2016, 2017, and 2019, numerous researchers have been proposed good research related to SA using machine learning, ensemble learning, and hybrid techniques.

#### Publication platform for SA

The total number of 92 documents from 2010 to 2022. We found 48 different journals for SA publications. The publications frequently occurred more than one time in our collection are reported in Table 6. Various conferences have also been scheduled for SA publication. The journals "Elsevier" and "Springer" are two more common venues for SA publications. Where Elsevier, Springer, and ACM are three popular publishers that are chosen by researchers for SA-related authentic research. Additionally, it has been seen that several platforms are open for SA-related research.

**Table 6:** Frequently used platforms for SA publication set (N = 92)

Publication Platform	Frequency
Conference and workshop	19
Applied intelligence (Springer)	4
Expert systems with applications (Elsevier)	5
ACM SIGAPP applied computing review (ACM)	3
Decision support systems (Elsevier)	4
arXiv preprint	2
Procedia computer science (Elsevier)	2
Social network analysis and mining (Springer)	4
Other international journal	3

#### Popular datasets for SA

**Fig. 4:**
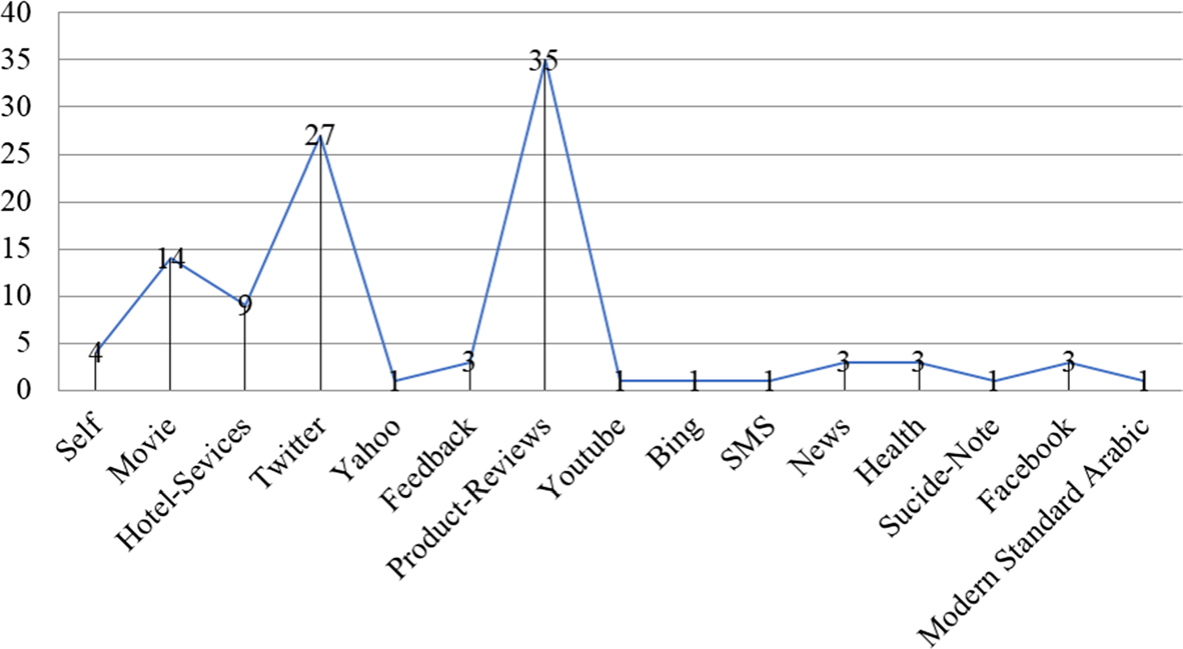
Popular datasets used for SA

A benchmark dataset plays a vital role in sound research. Figure 4 presents benchmark datasets used by researchers for SA. The researchers have used several resources, namely movie reviews, product reviews, Facebook posts, and tweets for SA. It has been seen in the graph that researchers more frequently use product reviews for their experiments. Secondly, Twitter gained more popularity among researchers for SA-related experiments. Few researchers also generate their datasets for sentiment classification. Whereas the researchers also consider movie reviews, medical reviews, and hotel reviews for SA-related experiments.

#### More favorable techniques for SA

This section provides knowledge about more frequently implemented methods for SA-related problems. Additionally, we explored more popular techniques related to the lexicon-based, machine learning-based, ensemble-based, graph-based, and hybrid-based approaches for SA applications. Table 7 presents the frequently used machine learning algorithms by researchers for the SA process, where SVM is in the topmost position in the list. NB was also persistently used by the researchers, but SVM produced the most noticeable results for sentiment classification.

**Table 7:** Frequently implemented machine learning techniques for SA set (N = 17)

Technique	Frequency
Support vector machine (SVM)	12
Naïve bayes (NB)	9
Maximum entropy (ME)	3
Tree	3
K-nearest neighbors (KNN)	2
Artificial neural network (ANN)	1
K-Star (K*)	1

Table 8 shows the frequency of ensemble-based techniques used for SA. It is observed that bagging and boosting are more common techniques researchers use for ensemble sentiment learning. The concept of majority voting has also frequently been implemented by researchers in different combinations of single learners.

**Table 8:** Frequently used ensemble techniques for SA set (N = 21)

Technique	Frequency
Bagging & boosting	15
Majority voting	11
Stacking	1

Table 9 presents the graph-based techniques for SA. There are many variations in choosing graph-based methods for SA, but word-graph and co-occurrence graph were used by two researchers in N set = 10.

**Table 9:** Frequently used graph-based techniques for SA set (N = 12)

Technique	Frequency
Word-graph	2
Co-occurrence graph	2
N-Graph	1
Rank algorithm	1
POS-based bigram algorithm	1
Random walk algorithm	1
Enterprise knowledge graph	1
Hyper-graph	1

A hybrid SA is very much in demand. So, we also surveyed various papers related to hybrid SA and categorized the hybrid work into five significant categories presented in Table 10. We found that in most of the hybrid work, researchers applied the combination of the lexicon approach and machine learning approach as it has been applied seven times in N set = 19. Whereas machine learning has been individually used five times, and a combination of machine learning and rule-based approaches has been used four times in previous work (N set = 19). The combination of machine learning with genetic and deep learning was found to be very rare.

**Table 10:** Frequently used hybrid techniques for SA set (N = 21)

Technique	Frequency
Combination of lexicon with machine learning	7
Machine learning	5
Combination of rule-based with machine learning	4
Combination of machine learning with genetic	2
Combination of machine learning with deep learning	1

## Important aspects of SA

SA has been an exciting field of study since the 1990s; there are further various sub-fields for research. Merriam-Webster defined sentiment as a thought, judgment, or attitude that arises from feeling. It is an idea or opinion developed by emotions. This section presents the various essential aspects of SA.

### SA challenges

SA is an emerging field, but it has various challenges, making it process-critical and decreasing the efficiency of related models. Although researchers are working to solve these issues using discriminant techniques, there is still a lack of accuracy. These challenges generate obstacles to extracting the correct meaning of sentiments and classifying the correct polarity. Common challenges of SA are mostly related to the language used in online social networks. Additionally, the words that regularly pronounce around us influence the words applied on online platforms. It is also noticeable that language used on social media is more malleable than formal words, including formal, informal, and personal communication language. Overcoming the mess of languages requires powerful natural NLP and linguistic skills. Table 11 presents the utmost challenges related to SA and proposed solutions.

**Table 11:** SA-related challenges and their proposed solutions

Challenge	Description	Solution
Stance-detection	Stance-Detection is an automatic process to check the favorability of text towards a given target. Most of the time, a bit of relevant information is not present in a review, which makes this a challenging task. The system first detects the target of a text and then identifies its implication of interest	Mohammad et al. (2016) created the first dataset of stance sentiment labeled tweets. Augenstein et al. (2016) experimented with conditional LSTM encoding to build a tweet representation dependent on the target. Sun et al. (2018) presented a neural model to employ linguistic information to construct document representation stance detection
Sarcasm-detection	Sarcastic sentences are those sentences that have the exact opposite meaning to write. It is complicated to extract the sarcastic sense because different people use different ways to show their sarcastic way. For example, "This movie is good enough to waste money” (Cabral and Hortacsu 2010). A conditional approach was applied to extract the sarcastic meaning from the text. Sarcasm detection becomes a big challenge in SA. However, various researchers are very active in this field	Bouazizi et al. (2016) proposed four types of features, namely "Sentence-Related" features, "Punctuation-Related” features, "Syntactic and Semantic" features, and "Pattern-Related" features to handle sarcastic tones differently. Joshi et al. (2015) presented word-embedding-based features and provided improvements over the previous four reported features
Negation-handling	Extracting the negation-affected patterns from sentences is a difficult task in SA. Negation handling is the process of reversing the polarity of phrases, words, or sentences where the scope of affected terms is not fixed. For example, "The movie was not entertaining," the scope was single after the negation word "not." Still, in another sentence, "I do not call this movie entertaining", the scope is not limited after the negation word "not". So, this is a challenging task for a system in SA	Farooq et al. (2017) proposed a linguistic feature-based negation handling method to determine the effect of various negations. Gautam et al. (2018) proposed the LSTM-based linguistic negation handling model
Domain-dependence	Domain-Dependence is a big issue in SA. Once a lexicon has trained for a particular domain, it mostly does not appropriate for other fields. Therefore, various types of research have been done to solve the problem of domain dependence in SA	Pan et al. (2010) proposed a Special Feature Alignment (SFA) algorithm to align domain-specific words that combined the words of different domains into a single cluster. Peng et al. (2018) proposed a simultaneous domain extraction method and introduced a few labeled target domain data to learn domain-specific information. Du et al. (2020) provided an effective way to use the pre-trained BERT language model to handle the domain dependence problem and also proposed a novel domain-distinguish task for pre-training
Huge-Lexicon	Every day, many reviews, opinions, news, and blogs are posted on social media sites that contain a huge vocabulary, and handling this huge vocabulary (lexicon) is a very complex task in SA	Kaushik et al. (2014) performed the SA using the Hadoop technique to handle many datasets
Word-sense disambiguation	Disambiguation is a problem identifying a word's sense because a single word can have discriminant meanings. The context of a sentence can control word sense disambiguation. For example, if the word "small" relates to the house, it reflects a negative sense, but it represents a positive sense for pet animals or other things. So, this is a challenging task for a system	Yu et al. (2003) and Hu et al. (2004) initiated a lexicon dictionary where words were linked by prior polarity context. The presented polarity of a word in a phrase may differ from the preceding polarity of words because a word can reflect different meanings
Anaphora resolution	Although "Pronouns" play an essential role in accurate sentiment extraction, they are ignored most of the time. For example, "The television is good. It has a big screen and good resolution". In this example, television cannot refer to good without knowing the pronoun 'it', leading to the problem of anaphora resolution	Ge et al. (1998) incorporate various factors of anaphora resolution into the specific statistical framework and maps the distance between the pronouns and proposed antecedent. Lappin et al. (1994) proposed an algorithm to identify precursors of third-party pronouns and lexical anaphors

### SA feature engineering

**Fig. 5:**
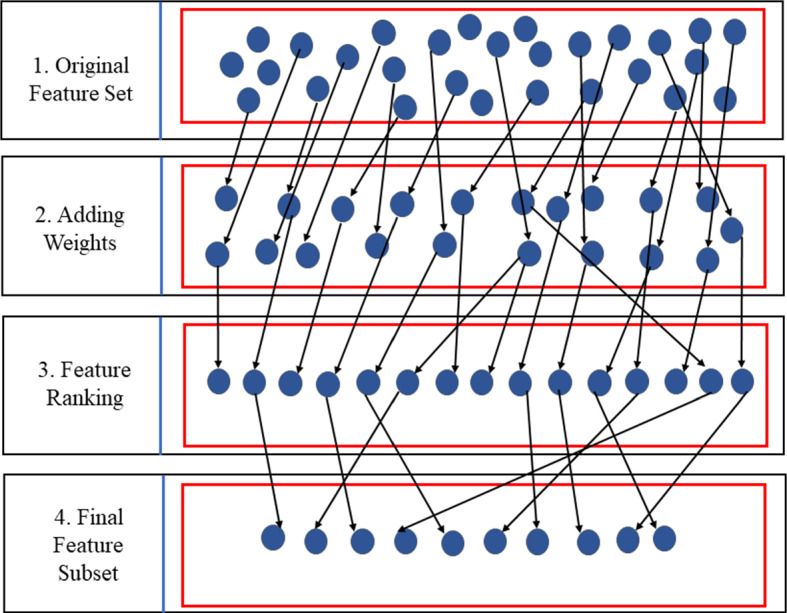
Feature engineering process

The number of N features increases the domain dimensionality of the datasets. Feature engineering is a very important step in SA applications and opinion mining. Feature selection and feature extraction should be intractable with final processing in optimal feature engineering (Kohavi and John 1997). This section provides information regarding various types of feature engineering techniques that have been previously applied for text preprocessing. Figure 5 depicts the process of feature engineering that completes in four steps: (1) Original Feature Set: This section holds the raw elements of the dataset that needed processing. (2) Adding Weights: All the calculations are performed, and weights are assigned to the selected features by normalization and scaling methods. (3) Feature Ranking: It is the process of arranging the features in specific order by the value of some scoring function. (4) Final Feature Subset: This represents the finally selected N number of features ready for the fact calculation (Liu et al. 2020).

Dimensionality reduction reduces the high dimensions of the dataset that keeps more discriminative and constructive features from the collection set. Feature engineering is categorized into two major parts (1) Feature Extraction and (2) Feature Selection. Feature extraction is a process of selecting required or essential features from the original set. Principal Component Analysis (PCA) and Latent Semantic Analysis (LSA) are the two popular techniques of feature extraction (Zareapoor and Seeja 2015). At the same time, feature selection is a process that reduces the number of variables for predictive models. Effective and efficient feature selection improves the performance of SA. The feature selection process includes missing values removal, low variance removal, highly correlated feature removal, univariate selection, and recursive elimination. Feature selection methods are categorized into two groups: filtered methods and wrapper methods (Uysala and Gunal 2014).

**Fig. 6:**
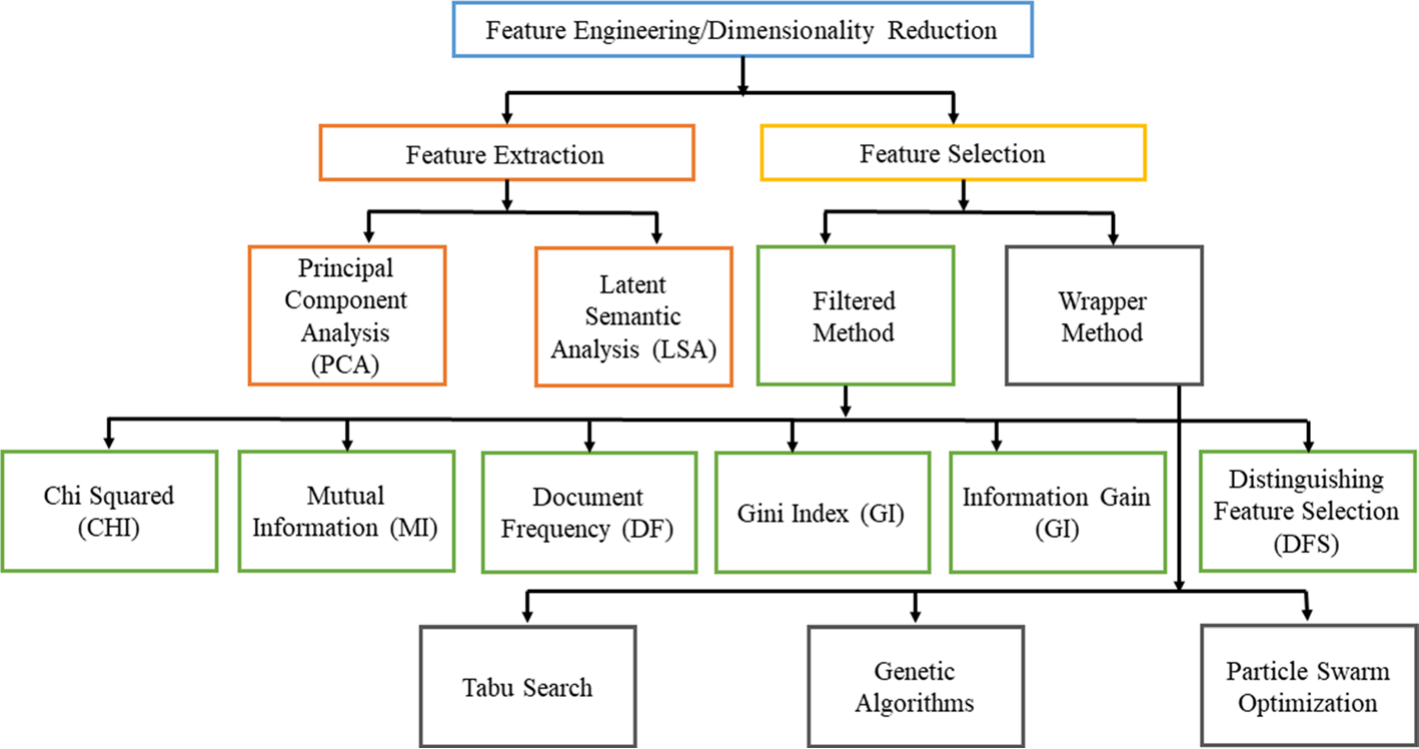
Feature engineering taxonomy

At the same time, feature selection is a process that reduces the number of variables for predictive models. Effective and efficient feature selection improves the performance of SA. The feature selection process includes missing values removal, low variance removal, highly correlated feature removal, univariate selection, and recursive elimination. Feature selection methods are categorized into two groups: filtered methods and wrapper methods (Uysala and Gunal 2014). Filtered methods do not depend on learning models or classification algorithms and can easily apply quickly. Chi-Squared (CHI), Mutual Information (MI), Document Frequency (DF), Gini Index (GI), Information Gain (IG), and Distinguishing Feature Selection (DFS) are the filtered feature selection methods. In contrast, wrapper methods depend on learning models and follow the rules accordingly. Tabu Search, Genetic Algorithms, and Particle Swarm Optimization (PSO) are the wrapper feature selection methods. Figure 6 presents the taxonomy of feature engineering/dimensionality reduction.

#### Feature extraction

In the SA task, the reviews and documents hold million and billion of tokens that make the text classification process more complex. Feature extraction is a dimensionality reduction method that reduces the N number of dimensions from the dataset and presents it in a more predictive and compact way (Gomez et al. 2012). The reduced set is easier to handle due to its size and contains only essential features for the process.

##### PCA

It is a popular technique to reduce the dimensionality of the dataset by converting the original attribute into a smaller unit. The purpose of the PCA has to derive new variables from the combinations of actual variables. PCA identifies the patterns in a dataset based on the correlation among various features.

1 $$\mu = \frac{1}{m}\sum\limits_{i = 1}^{m} {X^{i} }$$

2 $$\sigma_{j}^{2} = \frac{1}{m}\sum\limits_{{}}^{{}} {(X_{j}^{i} )^{2} }$$

3 $$\sum = \frac{1}{m}\sum\limits_{i = 1}^{m} {(X^{(i)} )(X^{(i)} )^{T} }$$

First, the mean of each feature is calculated in [Eq. 1] (Kumar et al. 2017). The mean vector of the column vector $\mu$ is $N \times 1$.To treat the different attributes as on the same scale, rescaling of each coordinate has been done to get a unit variance [Eq. 2], then replace $X\left( i \right)$ with $X\left( i \right) \, / \, \sigma j$. After completing the preprocessing, the covariance matrix has calculated using eigenvectors with the symbol $\sum$ (Greek letter sigma) [Eq. 3].

##### LSA

LSA is the latest dimensionality reduction technique and feature extraction in text classification. LSA works on the procedure of analyzing concepts, terms, and relationships between unstructured texts. It can correlate semantically related terms of latent text. LSA is used for text clustering and page retrieval system. LSA resolves the problem of words with more than one meaning and various words containing similar meanings (Zareapoor and Seeja 2015).

#### Feature selection

Feature selection, select and remove irrelevant and duplicate attributes from the dataset that do not contribute to the predictive model's performance and accuracy. Feature selection contributes to improving the model's performance, develops cost-effective predictors, and provides more simple and reliable models. Feature selection is a powerful tool to simplify or speed up the calculations of the learning model (Dasgupta et al. 2007). Feature selection is further categorized into filter method and wrapper method.

##### Filtered method

This method allows diverse scoring techniques to access the relevancy of features independently from learning classifiers or models. These techniques are very scalable to high dimensions datasets and provide fast and simple computations (Guyon and Elisseeff 2003). Various filter methods are available for text classification and SA.

- *CHI* It is a popular statistical method of feature selection that estimates the feature independently by calculating the chi-square corresponding to the class. It analyses the dependency between the term and class. It calculates 0 for the independent relationship and 1 for the dependent relationship between term and class (Zareapoor and Seeja 2015). CHI provides the significance difference formation and provides the significance difference information between categories (McHugh 2013).
  4 $$CHI(t,c_{i} ) = \frac{{N*(AD - BE)^{2} }}{(A + E)*(B + D)*(A + B)*(E + D)}$$
  5 $$CHI_{max} (t) = max_{i} (CHI(t,c_{i} ))$$
  The CHI [Eqs. 4] and [5] calculate the association between the features of the word and the associated class (Sharmac and Dey 2012). Here, $A$ represents frequency when $t$ = term and $c_{i}$ = class co-occur, $B$ is a count while $t$ appears without $c_{i}$, $E$ means events while $c_{i}$ appears without $t$, $D$ represents frequency while neither $c_{i}$ nor $t$ appears, and $N$ shows entire documents of the corpus. The score of CHI will be 0 when $t$ and $c_{i}$ are not dependent on each other.
- *MI* MI presents the association or dependence between the two random variables. MI finds the dependence between term t and class c. It describes the amount of information contained by a term for the associated class [Eq. 6] (Yang and Pedersen 1997). It is calculated as:
  6 $$MI(t,c) = log\frac{P(t|c)}{{P(t)}}$$
  Here, $P$ represents the probability of term $t$, and $P(t|c)$ represents the probability of term $t$ of assigned class $c$. MI measures the much information is communicated on average from one random variable to another. $P(t)$ and $P(c)$ are the marginal distribution of $t$ and $c$ get through the marginalization process.
- *DF* This threshold is the most straightforward technique to reduce the vocabulary of text classification. It can easily scale the massive corpora with the linear computational complexity of training documents. It does not recommend an extemporary approach as a principled criterion for feature selection. DF represents the number of documents in which a term appears. DF follows the assumption that infrequent terms are non-descriptive for the predictions of categories (Yang and Pedersen 1997). This method continuously removes those features whose frequency has greater or less than the predefined threshold.
- *GI* It is an improved version of the attribute selection algorithm used for feature selection (Alper Kursat Uysalab 2016). It works as a split measure for selecting the most appropriate splitting attribute in the decision tree. The simple formula is utilized to calculate the GI [Eq. 7].
  7 $$GI(t) = \sum\limits_{i = 1}^{M} {P(t|C_{i} )^{2} P(C_{i} |t)^{2} }$$
  Where, $P\left( {t|C_{i} } \right)$ shows the probability of term $t$ for class $C_{i}$, $P\left( {C_{i} |t} \right)$ shows the probability of $C_{i}$ presence in term $t$. $M$ represents the number of class labels and $P$ shows the proportion of $i^{th}$ class label. So, GI is the measure of anti-homogeneity hence the feature of minimum impurity is selected for the best feature split.
- *IG* It is a feature selection technique that reduces the size of features by computing and ranking the value of attributes. It measures the presence and absence of information in terms of contributing accurate classification. IG provides a higher score to those terms that hold relevant information for text classification.
  8 $$IG(t) = - \sum\limits_{i = 1}^{M} {P(C_{i} )logP(C_{i} ) + P(t)\sum\limits_{i = 1}^{M} {P(C_{i} |t)logP(C_{i} |t) + P(\mathop t\limits^{ - } )\sum\limits_{i = 1}^{M} {P(C_{i} |\mathop t\limits^{ - } )logP(C_{i} |\mathop t\limits^{ - } )} } }$$
  It is a global feature selection metric that calculates only one score for a particular term [Eq. 8] (Alper Kursat Uysalab 2016). Where, $M$ represents a number of classes, $P\left( {C_{i} } \right)$ probability of class $C_{i}$, $P(t)$ and $P\left( {\overline{t} } \right)$ shows probabilities of term $t$ presence and absence, $P\left( {C_{i} |t} \right)$ and $P\left( {Ci|\overline{t} } \right)$ are the conditional probabilities of class $C_{i}$.
- *DFS* It is the latest feature selection method and global metric for text classification. DFS selects distinguish features from the collection of sets and eliminates ambiguous ones based on predefined criteria [Eq. 9] (Uysalc and Gunal 2012).
  9 $${\text{DFS(t) = }}\sum\limits_{{\text{i = 1}}}^{{\text{M}}} {\frac{{{\text{P(C}}_{{\text{i}}} {\text{|t)}}}}{{{\text{P(}}\overline{{\text{t}}} {\text{|C}}_{{\text{i}}} {\text{) + P(t|}}\mathop {\text{C}}\limits^{{\text{\_}}}_{{\text{i}}} {) + 1}}}}$$
  Where, $M$ represents total classes, $P\left( {C_{i} |t} \right)$ shows the conditional probability of class $C_{i}$ in the presence of term $t$, $P\left( {\overline{t} |Ci} \right)$ presents the conditional probability of the absence of $t$ in $C_{i}$, $P\left( {t|\overline{C}_{i} } \right)$ represents the conditional probability of $t$ for all classes except $C_{i}$.

##### Wrapper method

The wrapper method uses a specific learning rule for feature selection tasks. The calculation cost of the wrapper method is high, and processing is slow. Wrapper methods are not usually preferred in SA and text classification due to their high price and slow performance (Baccianella et al. 2013). Wrapper methods are based on optimization concepts and intuitive search. Wrapper methods are used to find better features and reduce duplicate elements using cross-validation (Inza et al. 2004).

- *Tabu Search* It integrates learning techniques to evaluate only promising feature subsets. Tabu search generates better accuracy than a genetic algorithm, heuristic search algorithm, PSO, and an evolutionary search for text classification (Alper Kursat Uysald 2018).
  10 $$Accuracy = \frac{number \, of \, well \, classified \, observations}{{total \, number \, of \, observations}}$$
  11 $$features = 1 - \frac{\# S + Features}{{\# Features}}$$
  Most of the classification calculates the accuracy, which is calculated in Tabu search [Eq. 10] (Mousin et al. 2016). After that, to get a more interpretable learning model, the selected feature should minimize [Eq. 11].
- *Genetic Algorithms (GA)* GA is an optimal random search-based feature selection method that works on the propaganda of biological science mechanisms. It follows the procedure of genetic evolution in biology that starts from the initial feasible population and after that, applies crossover and mutation (Lei 2012). GA is a promising way to handle conditional optimization problems and is used immensely for feature selection.
- *PSO* It is used to select the most optimal feature from the collection set that provides the most remarkable difference between metallic particle classes in terms of their dimensions. PSO offers various advantages for powerful exploration. PSO has memory, inexpensive computation capability, potential population solution, address binary and discrete data, better performance, and is unaffected by dimension problem, which makes it an optimized and promising feature selection algorithm (Sharkawy et al. 2011).

### SA emotion theories

Emotion extraction and classification are essential parts of SA. So, here we introduce some types of basic emotions considered by the researchers in SA and classification. Here, we introduced a standard emotion set that is common in various research. Automatic human facial expression extraction is an emerging application of Human–Computer Interaction (HCI) and affective computing. Therefore, emotion extraction and classification became prime aspects in the research field of SA. Several researchers have been working on a distinctive set of emotions and expressions.

**Fig. 7:**
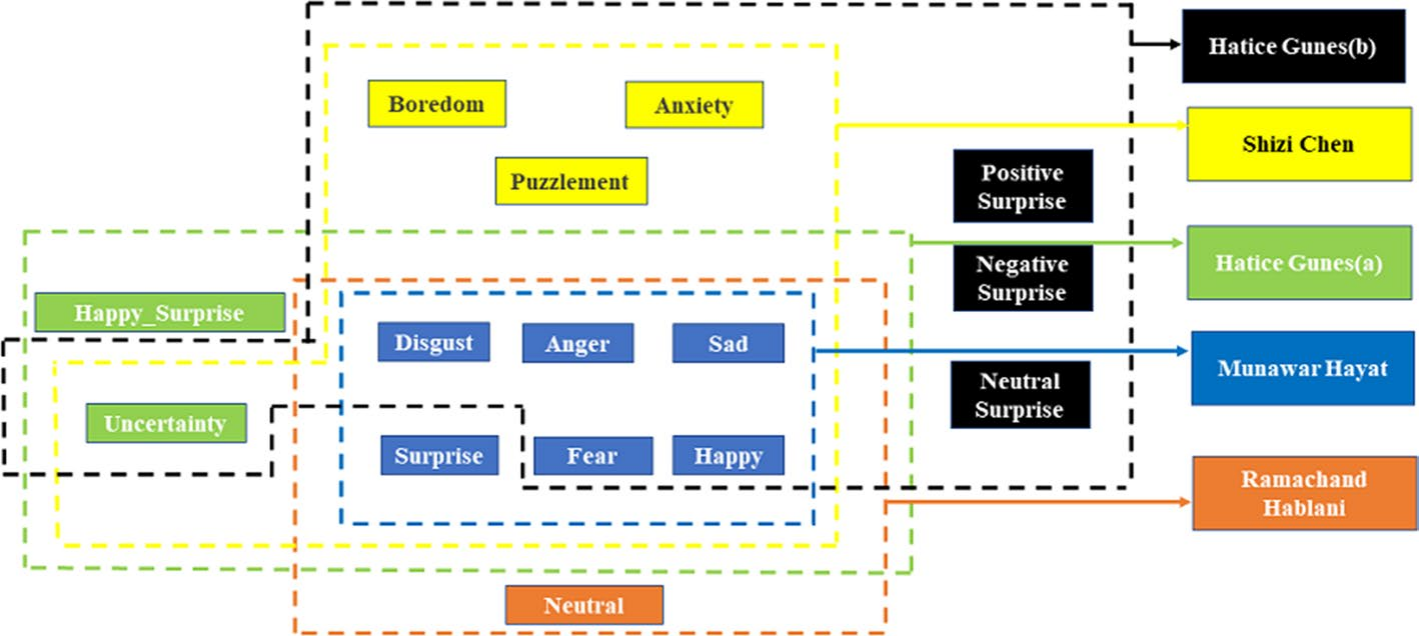
Taxonomy of frequently used emotion sets by researchers

Gunesa et al. (2005) present automatic emotion recognition from the face and body using early fusion and late fusion approaches. Their study performed on eight prototypical expressions; disgust, fear, anger, sad, happy, surprise, happy surprise, and uncertainty. Gunesb et al. (2008) used twelve emotions: disgust, fear, sadness, happiness, anger, uncertainty, anxiety, positive surprise, negative surprise, neutral surprise, boredom, and puzzlement for facial expression and body gesture extraction. Hablani et al. (2013) evaluated binary patterns for facial recognition of a person and classified their expressions according to seven basic emotions; disgust, fear, anger, sadness, happiness, surprise, and neutrality. Chen et al. (2013) used appearance and temporal motion features for facial and body gesture recognition. They classified the emotions into ten categories: disgust, fear, anger, sadness, happiness, surprise, anxiety, boredom, puzzlement, and uncertainty. Hayat et al. (2014) presented an automatic facial recognition framework with six basic emotions: disgust, fear, happiness, anger, surprise, and sadness. Table 12 displays a few recent sets of emotions that the researchers frequently used and their findings regarding visual, motion, and sound effects. These sets of emotions will be helpful for beginners to proceed in emotion mining. Figure 7 provides a better illustration of the emotion sets used by the researchers in their facial recognition works. According to Table 11 and Fig. 7, "disgust, fear, happy, sad, anger, and surprise" are common emotions used by different researchers.

**Table 12:** Discriminant emotions set used by researchers

Author	Findings	Considered emotions	Types-of-data
Gunes^a^ et al. (2005)	The presented research provided the visual emotion recognition model that includes practical body gestures and facial expressions to identify the sentiments of people. It is stated that emotion classification using two modalities acquires better recognition results than individual facial modality. Therefore, a fusion of the features performs better than a fusion at the decision level	Disgust, Happy, Anger, Fear, Sad, Surprise, Neutral, Uncertainty, Happy Surprise	Visual
Gunes^b^ et al. (2008)	An automatic temporal segments and phases detection model is proposed that explores whether temporal phase detection can effectively support the recognition of affective states based on phase synchronization. It is stated that (1) body and face expressions are simultaneous but not strictly synchronous, and (2) explicitly temporal phase detection can improve the effect recognition accuracy	Disgust, Happy, Anger, Fear, Sad, Uncertainty, Anxiety, Positive Surprise, Negative Surprise, Neutral Surprise, Boredom, Puzzlement	Voice & Visual
Hablani et al. (2013)	The presented research evaluated binary patterns of facial expression recognition. Extensive experiments prove that person-dependent methods can produce more results than existing models	Disgust, Happy, Anger, Fear, Sad, Surprise, Neutral	Visual
Chen et al. (2013)	A novel model is invented that includes both Motion History Image (MHI)-Histogram of Oriented Gradients (HOG) and Image-HOG for affect recognition through the temporal normalization method. It has been stated that expression recognition with temporal dynamics produces better results than frame-based recognition	Disgust, Happy, Anger, Fear, Sad, Uncertainty, Anxiety, Boredom, Puzzlement	Visual & Motion
Hayat et al. (2014)	A fully automated framework has been presented to exploit the dynamics of texture 3D videos for recognizing facial expressions. An effective graph-based spectral clustering is utilized to separately cluster the different facial points	Disgust, Happy, Anger, Fear, Sad, Surprise	2D & 3D Visual

## Methodology used for comparative analysis

**Fig. 8:**
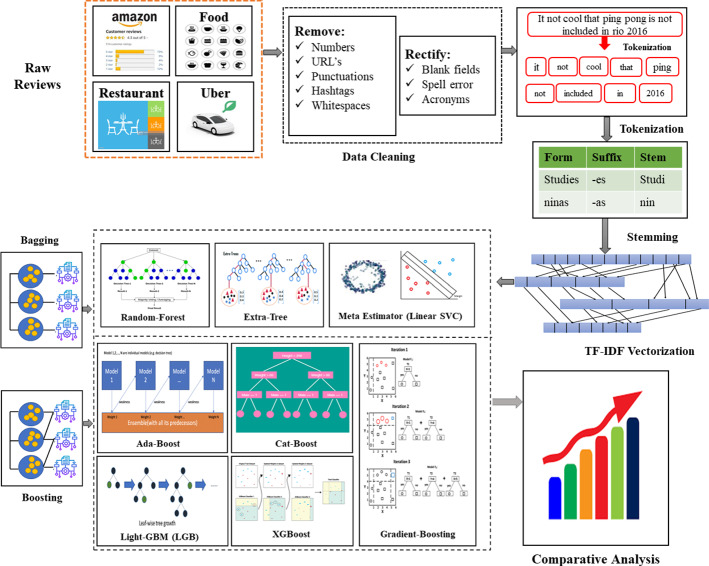
Methodology used for comparative analysis

This section presents the methodology used for ensemble classification of the text reviews for sentence-level sentiment classification. The ensemble approach of machine learning has been used in various applications and has produced outstanding results. Ensemble learning is also approachable in the SA task. Therefore, we have presented a comparative analysis of diverse ensemble methods that are divided into two main categories: bagging and boosting. This study compares eight popular ensemble learners (Random-Forest, Extra-Tree, Meta-Estimator (Linear SVC), Ada-Boost, Gradient-Boosting, XGB, Cat-Boost, and Light-GBM) to choose the best model for SA. The experiments have been conducted on four different domain reviews: Uber reviews, Restaurant reviews, Amazon reviews, and Food reviews. Figure 8 presents the comprehensive structure of the methodology used for comparative analysis. Further sub-sections provide detailed information regarding the comparative methodology.

### Dataset collection

Dataset collection is the initial step of every research, and it plays a crucial role in authentic experiments. Four leading review (Uber, Restaurant, Amazon, and Food) resources have been chosen to verify the authenticity of the experiments. Uber reviews dataset contains 1344 customer ride reviews, the Food category reviews dataset holds 25,000 records, Amazon product and Restaurant reviews dataset holds 1000 records for the experiment. Here, both large and small size of the dataset is collected for investigating the ensemble models that can provide better comparative analysis. The experimental dataset contains positive and negative reviews where positive sentiments are denoted by 1 and negative sentiments are denoted by 0. Table 13 displays the number of positive and negative reviews contain by all the datasets.

**Table 13:** Information regarding experimented datasets

Dataset	Total number of reviews	Total number of positive reviews	Total number of negative reviews
Uber reviews	1344	233	1111
Food reviews	25,000	12,500	12,500
Amazon Reviews	1000	500	500
Restaurant Reviews	1000	500	500

### Data preprocessing

It is required to convert raw data into a machine-understandable form. First, we organized the datasets by rectifying the spelling errors, antonyms, and missing fields. After that, basic steps such as punctuation removal, whitespace removal, URL removal, number removal, and hash-tag removal have been made to clean the reviews. These preprocessing steps are needed to get an accurate score for SA because machine learning cannot work effectively on raw and grubby datasets.

### Tokenization

Tokenization is a fundamental splitting phase in SA that partition the sentence, phrase, or paragraph into single words called tokens. Here tokens can be either character or word that is individually counted. Tokenization is the building block of NLP that is enforced by the n-gram approach. N-gram is a series of n items available in the text or speech. These can be categorized into unigrams, bigrams, or trigrams [Eq. 12].

12 $$ngramS = X - (N - 1)$$

where $X$ denotes the total number of words in the sentence $S$, and the value of $N$ will be 1 for unigram, 2 for bigram, and 3 for trigram. In unigram, sentences or phrases are split into the tokens of one word. In bigram, two words together are treated as a single token, and in trigram, three words together are treated as single tokens.

### TF-IDF vectorization

Vectorization is the process of converting text into meaningful, informative numbers. It measures the frequency of a word in a document and generates a number accordingly. TF is calculated by the number of times an individual word occurs in a document divided by the total number of words in a document.

13 $$w_{i,j} = tf_{i,j} \times \log\left (\frac{N}{{df_{i} }}\right)$$

IDF is used to assign the weights to rare words in the documents. TF-IDF is calculated in [Eq. 13]. Where $N$ represents the total number of documents, *tf*_*ij*_ is the total number of $i$ in $j$, and $df_{i}$ is the number of documents contained by $i$ (Term Frequency xxxx).

### Ensemble techniques

Machine learning supports two types of ensemble techniques bagging and boosting. Bagging selects the random samples from the training set and trains multiple learners Parallelly. In contrast, boosting collect the samples from the output of the previous learner and trains them sequentially. This section describes all the experimented ensemble techniques implemented for comparison. These algorithms are divided into two parts bagging and boosting. Wherefrom the bagging concept, we have selected a Random-Forest (RF), Extra-Tree (ET), and Meta-Estimator (Linear SVC) (M-SVC) for the implementation of SA, and from boosting approach, Ada-Boost (AB), Cat-Boost (CB), Gradient-Boosting (GB), XG-Boost (XGB), and Light-GBM (LGBM) were implemented.

#### Bagging ensemble approach

Bagging combines homogeneous classifiers and trains them parallelly with random samples. First, multiple bootstrap samples have been created that act individually. After that, base learners are fitted on them, and finally, their outputs are aggregated. Bagging is a popular ensemble approach that helps to reduce the variance of classifiers. Table 14 illustrates the procedure of the bagging ensemble approach (Polikar 2006).

**Table 14:**
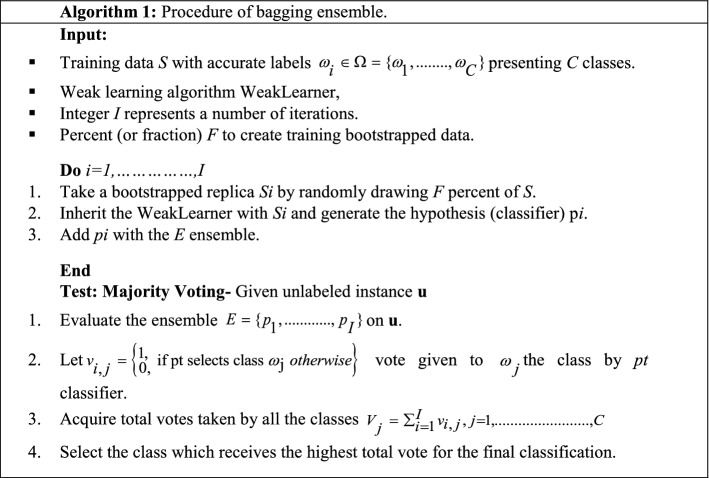
Process of the bagging approach

##### Random-forest

It is a powerful technique to handle large datasets quickly. Various applications have used it for accurate and effective results. Random-Forest constructs the multiple decision trees that classify the new instance by majority voting. Each node of the DT uses a randomly selected sample from the whole original sample set. We can say that every tree uses a different bootstrap sample, the same as the bagging concept. It follows a few steps:

14 $$ni_{j} = w_{j} C_{j} - w_{left(j)} C_{left(j)} - w_{right(j)} C_{right(j)}$$

15 $$fi_{i} = \frac{{\sum j:node \, j{\text{ splits on feature }}i^{ni}\, j}}{{\sum k \in {\text{all nodes}}^{ni} \,k}}$$

16 $$normfi_{i} = \frac{{fi_{i} }}{{\sum j \in all \, features^{{fi_{j} }} }}$$

17 $$RFfi_{i} = \frac{{\sum_{j \,in all \, trees} normfi_{ij} }}{T}$$

Equation 14 calculates the node importance of a tree. Where $ni_{j}$ represents the importance of node $j$, $w_{j}$ shows a weighted number of samples, $C_{j}$ shows the impurity value of node $j$, $left(j)$ is the left node, and $right(j)$ is the right node. Equation 15 calculates the importance of each feature on a decision tree. Where, $fi_{i}$ represents the importance of feature $i$. Equation 16 presents the normalization of these nodes. Finally, Eq. 17 shows the averaging method of all the trees. Where $RFfi_{i}$ shows the importance of feature $i$ calculated from all trees, $normfi_{i}$ represents normalized importance of feature for $i$ in tree $j$ and $T$ is the total number of trees (Random-Forest. xxxx).

##### Extra-tree

Highly Randomized Trees Classifier is an ensemble method that aggregates the output of multiple decision trees. It is highly similar to the random forest but only differs in DT construction in a forest. Table 15 presents the splitting process of the extremely randomized tree (Geurts et al. 2006).

**Table 15:**
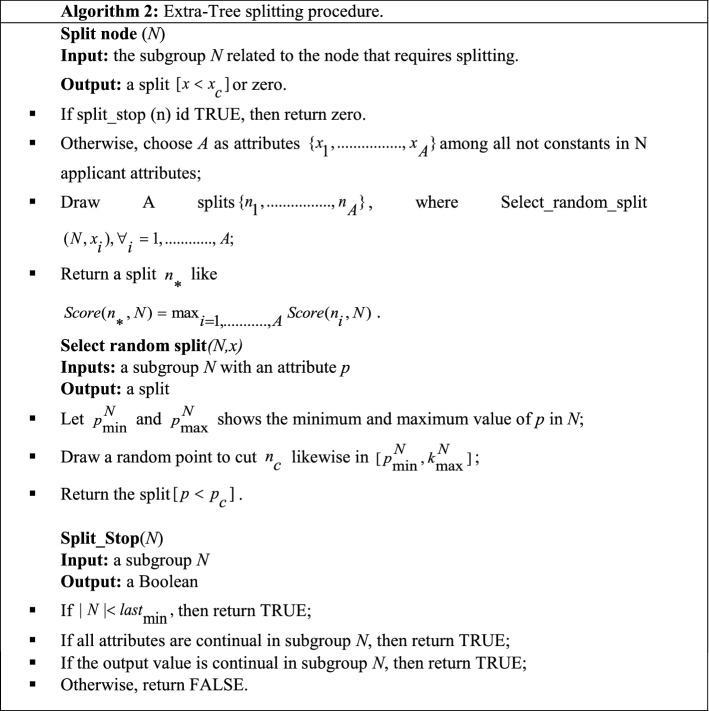
Splitting process of extremely randomized trees

Every DT of the Extra-Tree forest is formulated from the attributes of the original sample set. Then each individual node of the tree uses the random k feature of the sample, and each DT selects the best split for the creation of multiple de-correlated decision trees. Every DT calculates the entropy [Eq. 18] and information gain [Eq. 19].

18 $$Entropy(s) = \sum\limits_{i = 1}^{c} { - p_{i} \log_{2} (p_{i} )}$$

19 $$Gain(S,A) = Entropy(S) - \sum_{v \in Values(A)} \frac{|Sv|}{{|S|}}Entropy(S_{v} )$$

where, $c$ represents a number of labels (class) and $p_{i}$ is the proportion of rows. Extra-Tree classifier has simple properties, explicit meanings, and easy conversion of “if–then” rules (Sharaff and Gupta 2019).

##### Meta-estimator (linear SVC)

Bagging ensemble meta-estimator provides an option to select own base learner for the bagging process to reduce the base estimator's variance, e.g., a decision tree. Here, we have chosen Linear-SVC instead of DT as a base classifier for the bagging process. Linear SVC finds the hyper-plane space between two classes. It provides faster execution of large datasets and minimizes squared hinge loss. First, we built several substances of Linear SVC on random subsets of the original training set. After that, it aggregates the individual classified results of all substances to form a final classification.

#### Boosting ensemble approach

Boosting is an ensemble learning approach that boosts the performance of weak learners by sequentially running on multiple subsets of the dataset. Boosting constructs a sequence of models, and each model trains by considering the ambiguity of the previous model (Freund and Schapire 1996). Most ensemble techniques have identical statistics sets for training while boosting has different statistics training sets altered by previously trained models (Drucker et al. 1994). Table 16 presents the flow of boosting ensemble approach (Torelli and Menardi 2008).

**Table 16:**
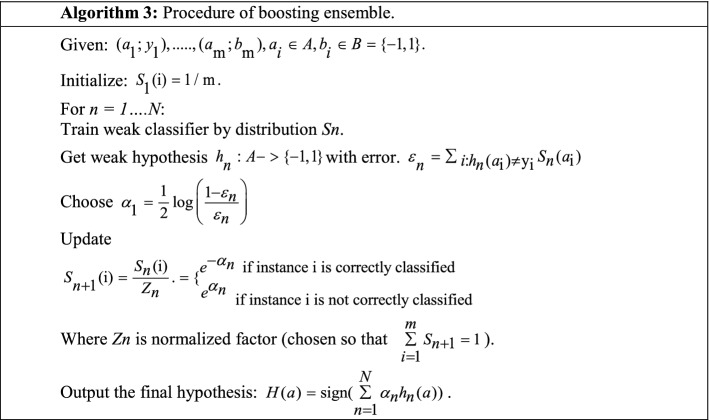
Process of the boosting approach

##### Ada-boost

It is the first boosting algorithm introduced by Freund and Schapire (1996) widely used in various applications. It boosts the performance of weak learners by converting them into stronger ones. Table 17 depicts the process of Ada-Boost learning (Bahad and Saxena 2020).

**Table 17:**
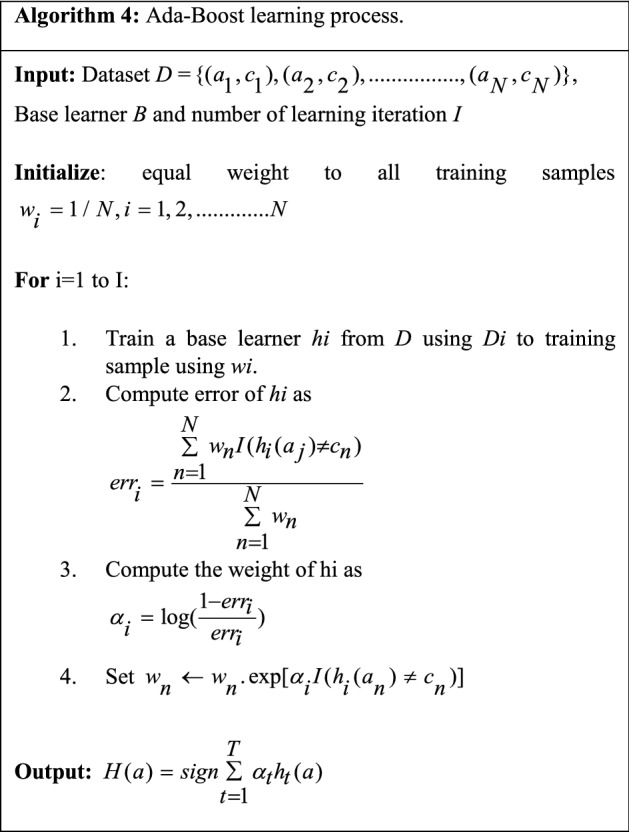
Learning process of Ada-Boost ensemble

Ada-Boost can train with any machine learning algorithm but is majorly applied with decision trees as these are very short and generate only one decision for classification. In this, trained models are sequentially added with weighted training data. Ada-Boost supports the concept of adaptive boosting, where weights are assigned to every instance, but higher weights are assigned to misclassified cases. The output is calculated as [Eq. 20].

20 $$F(x) = sign\left(\sum\limits_{m = 1}^{m} {\theta_{m} f_{m} (x)}\right )$$

where $f_{m}$ represents the $m_{th}$ weak classifier and $\theta_{m}$ is the assigned weight. It generates the weighted combination of $M$ weak classifiers.

##### Gradient-boosting

It is a powerful approach to building predictive models that generate additive models by statistically fitting parameterized functions to the current pseudo-residuals at each iteration of the model. The pseudo-residual is a gradient of the loss function that has been estimated on every present step. Respectively, at every iteration, a random subsample (without replacement) from the training dataset is drawn for base learning, which improves the execution speed and approximation accuracy of gradient boosting substantially (Friedman 2002).

21 $$F_{0} (x) = \gamma_{optimal} = \min \sum\limits_{i = 1}^{n} {L(y_{i,} \gamma } )$$

22 $$r_{im} = - \frac{{\partial L(y,F_{m - 1} (x))}}{{\partial F_{m - 1} (x)}}\left| {_{{{\text{x = x}}_{{\text{i}}} {\text{, y = y}}_{{\text{i}}} }} {\text{ f or all i = 1, 2, }}..{\text{, n}}} \right\rangle$$

23 $$D_{\bmod ified} = \{ (x_{i} ,r_{im} ):i = 1,2,......,n\}$$

24 $$\gamma _{m} = \arg \min \sum\limits_{{i = 1}}^{n} {L\left( {y_{i} ,F_{{m - 1}} \left( {x_{i} } \right) + \,\gamma h_{m} \left( {x_{i} } \right)} \right)}$$

25 $$F_{m} (x) = F_{m - 1} (x) + \gamma_{m} h_{m} (x)$$

First, a constant model has initialized with $F_{0}$ that fits on y-values [Eq. 21]. It begins with starting a constant model $\gamma$ with $\gamma \_optimal$ as an optimized problem. Second, Pseudo-residuals are calculated for each $i^{th}$ iteration [Eq. 22]. Where, $Fm - 1\left( x \right)$ represents the model derived by adding $m - 1$ weighted learners and primary persistent function. The rim represents the residual for the current base learner. Third fits a base learner $D\_\bmod ified$ on a derived subset of the training dataset [Eq. 23]. The fourth $\gamma_{m}$ multiplier is calculated by solving an optimization problem [Eq. 24]. The fifth $F_{m} \left( x \right)$ model has been updated and obtained a final model as $F_{m} \left( x \right)$ [Eq. 25].

##### Cat-boost

It is the latest ensemble technique that can incorporate deep learning techniques and work with discriminant data types to solve a wide range of problems. Cat-Boost is made with the combination of two words, "Category" and "Boosting," where category means it can work with varieties of data such as text, image, audio, or video, and boost means that it is a variant of gradient boosting ensemble. Cat-Boost resolves the exponential expansion of the feature combination generated by the greedy method at each split. Cat-Boost first divides the dataset into random subsets, then converts the labels into numerals, and finally transforms the category features into numbers [Eq. 26].

26 $${\text{avgTarget}} = \frac{countInclass + prior}{{totalCount + 1}}$$

Here, CountInclass represents a number of ones in the target for given categorical features; totalCount presents previous objects, and prior shows starting parameters (Meng et al. 2016). Table 18 presents the Cat-Boost learning process (Nguyen et al. 2018).

**Table 18:**
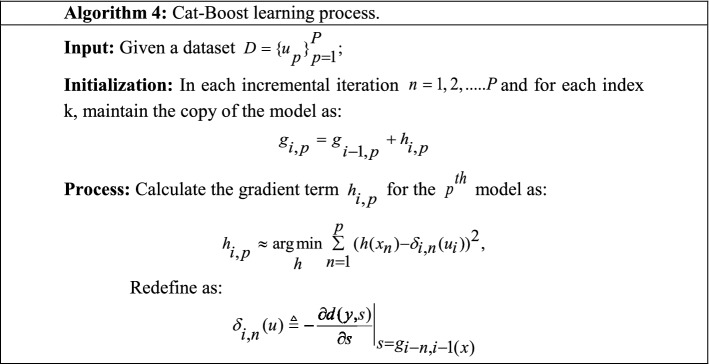
The learning process of the Cat-Boost ensemble

##### Extreme-gradient boost (XGB)

Tianqi Chen introduced XG-Boost to improve the performance of Gradient-Boosting. It includes a wide range of tools under the guidance of Distribute Machine Learning Community (DMLC) that can efficiently work with various interfaces. XG-Boost constructs different ensemble trees sequentially for ensemble learning and assigns weights to each value of the database, which decides the probability of getting selected for the next decision tree. The initial weight of each data value is the same, and it updates according to the further analysis of decision trees. The result obtained by the first DT helps to construct a new classifying model [Eq. 27], and this process is repeated repeatedly until the construction of the final model.

27 $$D = \{ (x_{i} ,y_{i} )\} (|D| = n,x_{i} \in R^{m} ,y_{i} \in R)$$

28 $$L(\phi ) = \,\sum\limits_{i} {l\left( {\widehat{y}_{i} ,y_{i} } \right)} \, + \,\sum\limits_{k} {\Omega \left( {f_{t} } \right)}$$

Here, $D$ is an ensemble model of a tree which applies $K$ additive functions [Eq. 28] to predict the output.

29 $$F = \{ f(x) = w_{q} (x)\} (q:R^{m} \to T,w \in R^{T} )$$

Here, $F$ in [Eq. 29] is a defined space, which is a part of regression trees, and $q$ presents the tree's structure.$T$ represents the number of leaves of a tree, and $f_{k}$ corresponds to the tree's structure.

30 $$L\left(\phi\right ) = \sum \limits_{i}^{{}} l\left({\widehat{{\text{y}}}_{i}} ,y_{i} \right) + \sum \limits_{k}^{{}} {\Omega (f_{k} )}$$

[Eq. 30] minimizes to provide information about the set of functions used in the model. The difference is measured between target $y_{i}$ and predicted $\text{y}i$.

31 $$L^{{(t)}} = \sum\limits_{{i = 1}}^{n} {l\left( {\widehat{y}_{i} ,\widehat{y}_{i} ^{{\left( {t - 1} \right)}} + \,f_{t} \left( {x_{i} } \right)} \right)\, + \,\Omega \left( {f_{t} } \right)}$$

[Eq. 31] presents the additive training process of the model.$f_{t}$ improves the model's accuracy by optimizing the objective, and $g_{i}$ in [Eq. 32] is second-order statistics related to the loss function.

32 $$L^{{(t)}} = \sum\limits_{{i = 1}}^{n} {\left( {l\left( {\widehat{y}_{i} ,\widehat{y}_{i} ^{{(t - 1)}} } \right) + g_{i} f_{i} \left( {x_{i} } \right) + \frac{1}{2}h_{i} f^{2} _{t} \left( {x_{i} } \right)} \right) + \Omega \left( {f_{t} } \right)}$$

33 $$L^{(t)} = \sum\limits_{i = 1}^{n} {\left( {g_{i} f_{t} (x_{i} ) + \frac{1}{2}h_{i} f^{2}_{t} (x_{i} )} \right) + \Omega (f_{t} )}$$

The constant function can also be removed for obtaining the following procedure presented by [Eq. 33]. This method is complicated in terms of depth. Hence, boosting trees generates high variance and low biased results. In contrast, random trees generate high bias and low variance in results because the model has a better ability to fit on the dataset (Bhati et al. 2020).

##### Light-GBM (LGBM)

It supports the Gradient-Boosting framework, which increases the efficiency of the model with light-weighted decision trees. It includes Exclusive Feature Bundling (EFB) and Gradient-based One Side Sampling (GOSS) techniques to overcome the limitation of the histogram that is primarily used by all Gradient-Boosting-based algorithms. Light-GBM is a variant of Gradient-Boosting, which inherits predictivity and resolves its scalability problem and long computational time using a leaf-wise growth scheme (Zhang et al. 2019). Light-GBM finds an approximation function to minimize the value of loss function [Eq. 34].

34 $$\widehat{f} = \arg \min E_{{y,X}} L\left( {{\text{y,}}\,{\text{f}}\left( {\text{x}} \right)} \right)$$

Then integrates the various $T$ regression trees for approximating the final model [Eq. 35].

35 $$f_{T} (X) = \sum\limits_{t = 1}^{T} {f_{t} (X)}$$

After that, Light-GBM trains in the form of additive approach at step $t$ [Eq. 36]

36 $$\Gamma_{t} = \sum\limits_{i = 1}^{n} {L\left(y_{i} ,F_{t - 1}\left (x_{i}\right ) + f_{t} \left(x_{i} \right)\right)}$$

In Light-GBM, the objective function is approximated continuously with Newton's method. The formulation is transformed in [Eq. 37] after removing the constant term in [Eq. 36].

37 $$\Gamma_{t} \cong \sum\limits_{i = 1}^{n} {\left(g_{i} f_{t} \left(x_{i}\right ) + \frac{1}{2}h_{i} f_{t}^{2} \left(x_{i}\right )\right)}$$

where $h_{t}$ and $g_{i}$ present first and second-order gradient statistics of the loss function. Let $I_{j}$ represents the sample set of leaf $j$ and [Eq. 37] transformed as [Eq. 38].

38 $$\Gamma _{t} = \sum\limits_{{j = 1}}^{j} {\left( {\left( {\sum\limits_{{i \in I_{j} }} {g_{i} } } \right)w_{j} + \frac{1}{2}\,\left( {\sum\limits_{{i \in I_{j} }} {h_{i} + } \lambda } \right)w_{j}^{2} } \right)}$$

For $q\left( x \right)$ tree structure, $w*j$ presents the optimal weight score of each leaf node and extreme value of could be formulated as [Eq. 39].

39 $$w*_{j} = - \frac{{\sum {_{{i \in I_{j} }} g_{i} } }}{{\sum {_{{i \in I_{j} }} h_{i} + \lambda } }}$$

40 $$\Gamma_{T}^{*} = - \frac{1}{2}\sum\limits_{j = 1}^{j} {\frac{{\left( {\sum {_{{i \in I_{J} }} g_{i} } } \right)^{2} }}{{\sum {_{{i \in I_{J} }} h_{i} + \lambda } }}}$$

Here is the scoring function that measures the quality of the tree $q$ structure [Eq. 40]. Finally, after adding the split objective function is as follows:

41 $$G = \frac{1}{2}\left( {\frac{{\left(\sum {_{{i \in I_{L} }} g_{i} } \right)^{2} }}{{\sum {_{{i \in I_{L} }} } h_{i} + \lambda }} + \frac{{\left(\sum {_{{i \in I_{R} }} g_{i} } \right)^{2} }}{{\sum {_{{i \in I_{R} }} } h_{i} + \lambda }} - \frac{{\left(\sum {_{i \in I} g_{i} }\right )^{2} }}{{\sum {_{i \in I} } h_{i} + \lambda }}} \right)$$

where $IL$ and $IR$ present the sample set of left and right nodes, respectively, Light-GBM trees grow vertically, unlike other Gradient-Boosting techniques, making Light-GBM more effective for processing the various features and large datasets.

## Comparative results

This section presents the comparative results of eight ensemble techniques (Ada-Boost, Gradient-Boosting, XGB, Light-GBM, Cat-Boost, Random-Forest, Meta-Estimator (Linear SVC), and Extra-Tree) on four popular reviews (Uber-Reviews, Restaurant-Reviews, Amazon-Reviews, and Food-Reviews) datasets. The experiments were conducted on a PC with Intel(R) Core (TM) i5-8265U processor, 4 GB RAM, 64bit operating system, and Windows-10 using Jupyter Notebook. All the datasets are partitioned into two parts, 80% for training purposes and 20% for the testing set. The standard measures, namely TPR, FPR, accuracy, weighted precision, weighted recall, weighted f1-score, AUC-score, and run-time, were adopted to check the performance of each ensemble model. The definition of all the employed measures is initiated with a confusion matrix, as presented in Table 19.

- *Accuracy* It is simply a ratio of accurate prediction to the total predicted observations [Eq. 42].
  42 $$Accuracy = \frac{TP + TN}{{TP + FP + FN + TN}}$$
- *Weighted Precision* It is a ratio of correctly positive predictions to the total positive predicted observations [Eq. 43].
  43 $$\Pr ecision_{Weighted} = \frac{{\sum\limits_{i = 1}^{m} {|y_{i} |\frac{{TP_{i} }}{{TP_{i} + FP_{i} }}} }}{{\sum\limits_{i}^{m} {|y_{i} |} }}$$
- *Weighted Recall* It is a ratio of correctly predicted positive observations to the total actual observations [Eq. 44].
  44 $${\text{Re}} call_{Weighted} = \frac{{\sum\limits_{i = 1}^{m} {|y_{i} |\frac{{TP_{i} }}{{TP_{i} + FN_{i} }}} }}{{\sum\limits_{i}^{m} {|y_{i} |} }}$$
- *Weighted F1-Score* It is a weighted average score of precision and recall [Eq. 45].
  45 $$F1 - Score_{Weighted} = \frac{{\sum\limits_{i = 1}^{m} {|y_{i} |\frac{{2TP_{i} }}{{2TP_{i} + FP_{i} + FN_{i} }}} }}{{\sum\limits_{i}^{m} {|y_{i} |} }}$$
- *ROC-AUC* It stands for the area under the Receiving Operating Characteristics Curve that measures the capability of classification technique to differentiate between the classes. A higher AUC score presents better classification, and a lower score shows inaccurate classification. The ROC-AUC curve plotted based on True Positive Rate (TPR) = TP/TP + FN on the x-axis and False Positive Rate (FPR) = FP/TN + FP on the y-axis (Bichitrananda Behera and Kumaravelan 2019).

**Table 19:** Confusion matrix

	Negativ	Positive
Negative	True Negative (TP)	False Positive (FP)
Positive	False Negative (FN)	True Positive (TP)

Table 20 reported the TPR, FPR, and run-time values of eight ensemble models. Accordingly, GB obtains the highest TPR value, 117.6, for Uber reviews. ET receives the highest TPR value, 60.19, for Restaurant reviews. M-SVC gets the highest TPR value, 68.26, for Amazon reviews, and CB obtains the highest TPR value, 88.70, for Food reviews. This shows that GB, ET, M-SVC, and CB are more capable than other ensembles of identifying the actual positives correctly. M-SVC scores minimum FPR of 0.0 and 12.75 for Uber reviews and Food reviews. In comparison, GB obtains a minimum FPR of 02.06 and 03.12 for Restaurant and Amazon reviews. In addition, M-SVC provides fast execution for small datasets, as it had taken the minimum time (97 ms and 67 ms) to run for Restaurant reviews and Amazon reviews datasets. Still, for the large Food reviews dataset, ET has taken a minimum of 2350 ms for execution. Conclusively, the M-SVC approach provides the highest TPR, lower FPR, and fast performance for text classification.

**Table 20:** Comparative results

Dataset	Measure	AB	GB	XGB	LGBM	CB	RF	M-SVC	ET
Uber reviews	TPR	52.94	117.6	41.17	32.35	35.29	38.23	38.23	35.29
	FPR	05.95	01.70	05.10	05.10	00.85	00.42	0.0	00.42
	Run-Time(ms)	1910	5220	2160	636	30,900	997	993	1400
Restaurant reviews	TPR	45.63	36.89	56.31	38.83	53.39	57.28	55.33	60.19
	FPR	07.21	02.06	13.40	17.52	10.30	16.49	14.43	16.49
	Run-Time(ms)	190	252	426	173	4220	286	97	340
Amazon reviews	TPR	55.76	38.46	65.38	50.00	62.50	67.30	68.26	67.30
	FPR	15.62	03.12	13.54	14.58	09.37	11.45	13.54	15.62
	Run-Time(ms)	1340	289	1370	682	3540	301	67	342
Food reviews	TPR	84.29	87.72	86.74	86.66	88.70	83.44	86.62	84.86
	FPR	23.94	47.64	16.48	15.46	15.81	14.56	12.75	13.14
	Run-Time(ms)	142,200	559,800	85,200	28,400	431,400	76,200	32,700	2350

**Fig. 9:**
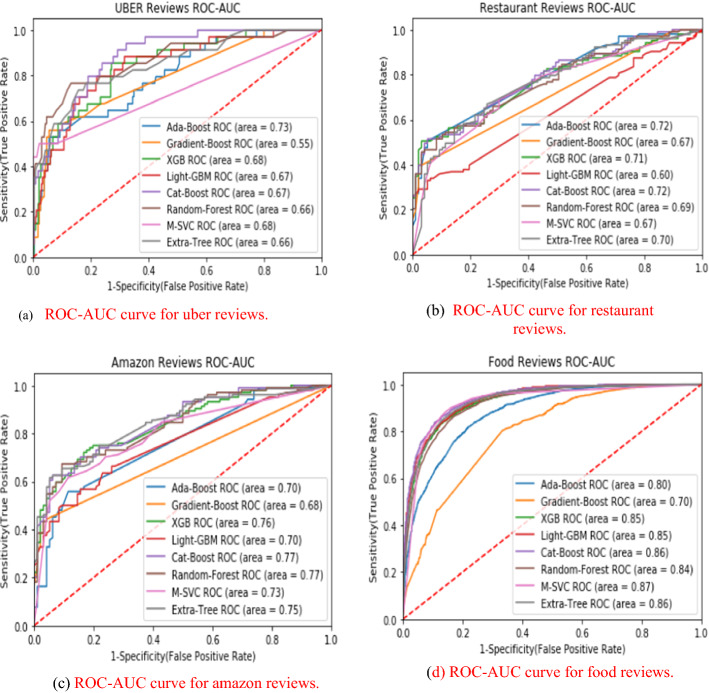
The combined ROC-AUC curve of ensemble models

Figure 9a, b, c, and d depicts the combined ROC-AUC score of experimented ensemble models for experimented datasets. It can be seen that Ada-Boost obtains the highest AUC score of 73 and 72 for Uber and Restaurant reviews datasets. Whereas Cat-Boost and Random-Forest score the highest AUC score, 77 for the Amazon reviews dataset. In the case of Food reviews, Meta-Estimator (Linear SVC) archives a higher AUC score of 87 for text classification. Ada-Boost obtains a higher AUC score for two (Uber and Restaurant) review datasets. We can say that Ada-Boost is the best model to classify text reviews. It has also been discovered that Meta-Estimator (Linear SVC) is more capable of classifying the reviews of the large dataset as it outperforms for Food reviews dataset, which stores maximum reviews.

**Fig. 10:**
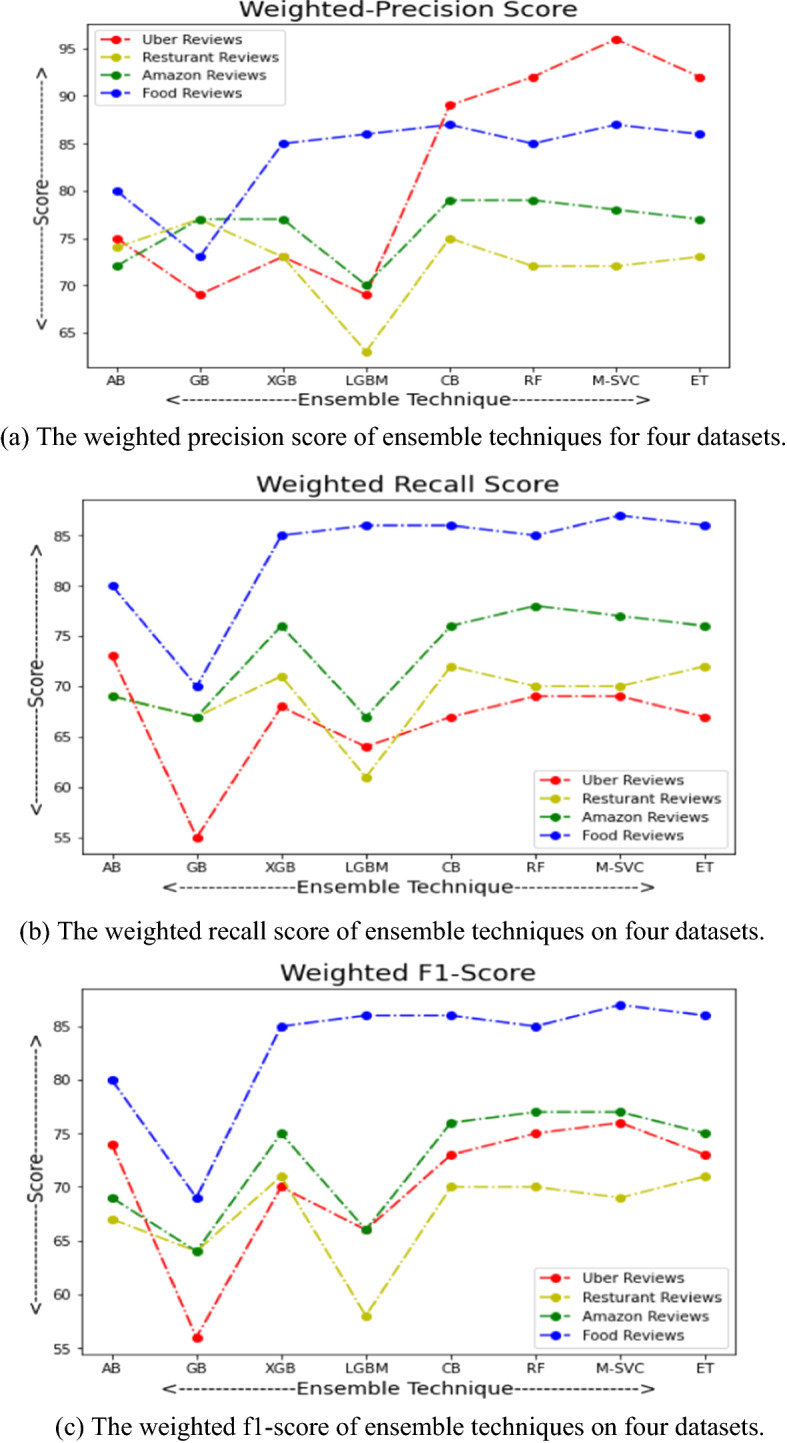
A comparative weighted precision, weighted recall, and weighted f1-score of ensemble techniques for four datasets

Figure 10 and Table 21 depict the weighted precision, weighted recall, and weighted f1-score of all the experimented models for four datasets. The bagging-based Meta-Estimator (Linear SVC) obtains a higher weighted precision value (93% and 87%) for the Uber and Food reviews datasets. The Cat-Boost and Random Forest ensemble achieves a higher weighted precision score of 79% for Amazon reviews. At the same time, XGB obtains higher weighted precision of 80% for Restaurant reviews. It means that Meta-Estimator (Linear SVC), Cat-Boost, and Random-Forest ensembles generate low false-positive rates to classify text, respectively. It can be seen that Meta-Estimator (Linear SVC) obtains higher weighted precision, weighted recall, and weighted f1-score of 87% for large Food review datasets, which indicates it is more capable of identifying actual facts and not disturbed by false rates correctly. Extra-Tree gives a higher weighted recall of 72% and a weighted f1-score of 71% for Restaurant reviews, and Random-Forest provides higher weighted precision of 79%, weighted recall 78%, and weighted f1-score 77% for the Amazon reviews dataset. Conclusively, from eight experimented ensemble techniques Meta-Estimator (Linear SVC), Random-Forest generates low false-positive and low false-negative rates for SA. Furthermore, Meta-Estimator (Linear SVC) is an efficient ensemble model for large and small datasets.

**Table 21:** Comparative weighted precision, weighted recall, and weighted f1-score of the ensemble models

Dataset	Measure	AB	GB	XGB	LGBM	CB	RF	M-SVC	ET
Uber reviews	Weighted-Precision	89%	84%	88%	87%	91%	92%	93%	92%
	Weighted-Recall	89%	87%	90%	88%	91%	92%	92%	91%
	Weighted-F1-Score	89%	84%	88%	87%	89%	90%	91%	90%
Restaurant reviews	Weighted-Precision	75%	78%	80%	63%	75%	72%	71%	73%
	Weighted-Recall	69%	67%	72%	59%	71%	70%	68%	71%
	Weighted-F1-Score	67%	63%	71%	57%	70%	70%	67%	71%
Amazon reviews	Weighted-Precision	72%	77%	79%	72%	79%	79%	74%	77%
	Weighted-Recall	69%	67%	74%	69%	76%	78%	73%	76%
	Weighted-F1-Score	69%	64%	74%	69%	76%	77%	73%	75%
Food reviews	Weighted-Precision	80%	73%	81%	85%	86%	85%	87%	86%
	Weighted-Recall	80%	70%	80%	85%	86%	85%	87%	86%
	Weighted-F1-Score	80%	69%	80%	85%	86%	85%	87%	86%

**Fig.11:**
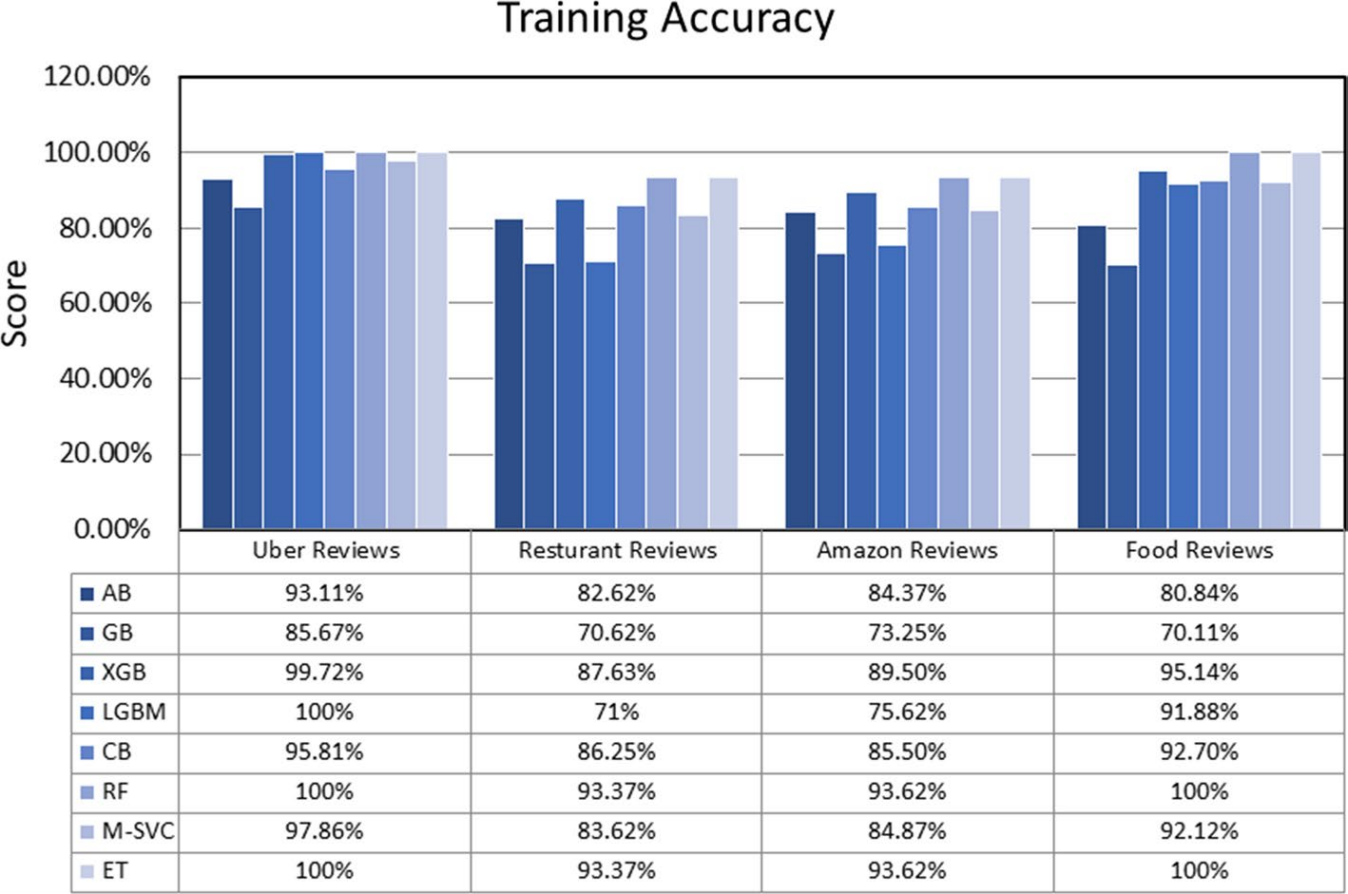
Training accuracy of ensemble models for different datasets

**Fig. 12:**
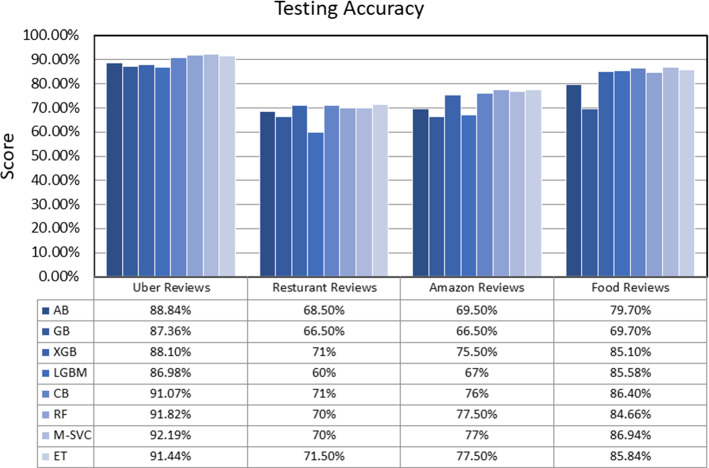
Testing accuracy of ensemble models for different datasets

Figure 11 depicts the training accuracy of experimented ensemble models for different datasets, and Fig. 12 presents the testing accuracy of tested ensemble models for other datasets. According to training accuracy, Extra-Tree and Random-Forest obtain higher and equal scores of 100% for Uber reviews, 93.37% for Restaurant reviews, 93.62% for Amazon reviews, and 100% for Food reviews.

In testing accuracy, Random-Forest achieves a higher score of 91.82% for Uber reviews, Extra-Tree achieves 71.50% for Restaurant Reviews, Random-Forest and Extra-Tree achieve a higher and equal score of 77.50% for Amazon reviews, and Meta-Estimator (Linear SVC) obtains 86.94% score for Food reviews. In addition, from the boosting concept, XGB receives a higher training accuracy score of 87.62%, 89.50%, and 95.14% for Restaurant, Amazon, and Food reviews datasets. The Cat-Boost ensemble obtains the highest testing accuracy score of 91.07%, 71.00%, 76.00%, and 86.40% for Uber, Restaurant, Amazon, and Food reviews datasets. For Uber reviews, Light-GBM obtains the highest and equal training accuracy of 100% with Random-Forest and Extra-Tree. Conclusively, Cat-Boost achieves better training and testing accuracy than all the boosting techniques but cannot beat the bagging approach's performance as Random-Forest and Extra-Tree outperform over boosting ensemble techniques.

After analyzing the results of all the experimented ensemble techniques according to the different measures, we discovered some important facts regarding the high and low performance of bagging and boosting-based ensemble models for SA using multiple datasets. We conclude the different types and lengths of datasets influence the performance of SA distinctly.

- Gradient-Boosting generates the minimum difference (1.69%, 4.12%, 6.75%, and 0.41) between training and testing accuracy scores for (Uber, Restaurant, Amazon, and Food) both large and small kinds of datasets, which means it overcomes the problem of overfitting and underfitting and reduces the bias and variance for training the model.
- Cat-Boost obtains state-of-the-art results for SA on discriminant datasets. It achieves higher testing accuracy and AUC score for all the experimented datasets. Cat-Boost is very easy to implement and generates competitive results with the help of one-hot encoding.
- As we know that Light-GBM is a robust algorithm and capable of handling large datasets but according to our experiments, Light-GBM provides less accuracy and AUC score for text classification than other experimented ensemble techniques. Although Light-GBM produces higher results as 91.88% training accuracy, 85.58% testing accuracy, and 85 AUC score for the large Food reviews dataset, still unable to beat the performance of Cat-Boost, and XGB.
- Cat-Boost and Gradient-Boosting are two main approaches with discriminant frameworks. Apart from it, XGB, Light-GBM, and Cat-Boost follow the framework of Gradient-Boosting. Experiments show Ada-Boost performs better than Gradient-Boosting in training, testing, and AUC scores for all the datasets but is unable to solve overfitting and underfitting problem, generating a higher difference between training and testing accuracy than Gradient-Boosting.
- Random-Forest and Extra-Tree are bagging-based approaches, where Random-Forest chooses the optimum split and Extra-Tree chooses random division for selecting the nodes. According to our experiments, both algorithms obtain equal training (100%, 93.37%, 93.62%, and 100%) accuracy on all the experimented datasets. For testing and AUC score, they also generate similar approximate values. Therefore, it can declare that Random-Forest and Extra-Tree algorithms are equally capable of sentiment classification.
- Meta-Estimator with Linear SVC is a bagging-based approach that uses Linear SVC for bagging procedures instead of decision trees. The demonstration shows that Meta-Estimator (Linear-SVC) obtains good results in terms of TPR, FPR, and run-time than all the experimented ensemble techniques, which means it can generate lower false positive and false negative rates and faster execution.

**Fig. 13:**
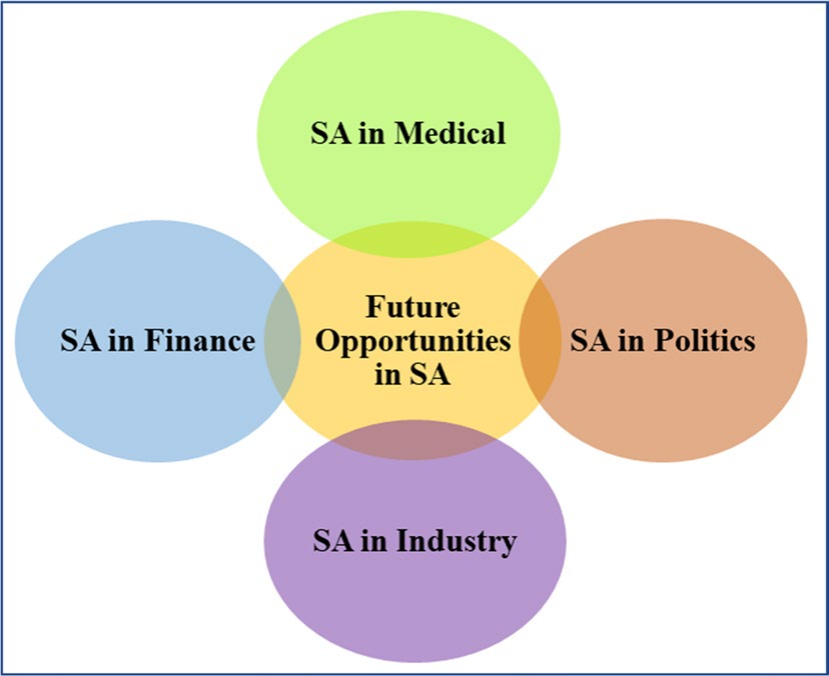
Future opportunities in the field of SA

As discussed above, our primary motive was to compare the bagging-based ensemble with the boosting-based ensemble to perform SA. After analyzing the results presented in Table 20, Figs. 10, 11, 12, and 13. We decide that bagging-based ensemble techniques (Random-Forest, Extra-Tree, and Meta-Estimator (Linear SVC)) performed better than boosting-based techniques. Random-Forest and Extra-Tree perform almost equally. Meta-Estimator (Linear SVC) gives less training accuracy and testing accuracy than Extra-Tree and Random-Forest but provides higher speed comparatively. However, XGB and Cat-Boost obtain better accuracy and TPR than other boosting ensembles but cannot beat the performance of bagging-based ensembles. Hence, bagging ensemble-based techniques provide state-of-the-art results for SA. In the introduction part, we have raised some questions regarding the essential aspects and trends of SA.

## Research opportunities in SA

SA has gained popularity in various fields, including medicine, politics, industries, and finance. Therefore, researchers are developing various intelligent models for SA. Figure 13 presents the major application areas for SA, where researchers can develop generalized frameworks for real-life applications. Further subsections describe these future opportunities of SA in detail.

### SA in medical

SA is gaining popularity in healthcare industries and improving the quality of healthcare services. The opinion and reviews of the patients help healthcare providers to diagnose a particular disease (Abualigah et al. 2020). The COVID-19 outbreak increased the demand for SA in healthcare-related services. SA has been applied to extract the opinion of people towards nation wise lockdown due to the COVID-19 pandemic (Barkur and Vibha 2020 Jun). A novel fusion model has been developed to study the tweets of various coronavirus-affected countries (Basiri et al. 2021 Sep). Medical documents reflect the information of the patients in terms of diagnosis, examinations, observations, and interventions. Judging the medical conditions of the patients in the form of positive and negative responses is required. Several methods have also been developed to handle these kinds of tasks (Denecke and Deng 2015 May 1). Therefore, health care departments needed huge research in the field of SA.

### SA in politics

In the current digital world, politics has moved on different levels, and countries' governments use social platforms to extract the people's opinions towards the established laws and policies. SA has been exponentially implemented to know the voice of people. A two-stage model has been developed to predict the results of the election (Ramteke et al. 2016). In the past two years, farmers' protests against three legislation bills passed by the Indian government have shaken the world. Here, artificial intelligence-based SA increased its demand to provide the direction for this democratic dispute (Neogi et al. 2021 Nov 1). A Twitter dataset of the US presidential election 2016 was collected and applied to the SA to find the choice of people between Hillary Clinton and Donald Trump (Somula et al. 2016). Hence efficient SA models have been required to solve political issues.

### SA in industries

SA provides huge support for incremental growth in businesses. Industries use various applications of SA, such as brand monitoring, feedback gathering, the voice of customers (VoC), product analysis, market research, and competitive research. These SA-based applications help industries with decision-making. A novel LSTM-CNN-based model has been developed using a grid search optimization method to find out the opinion of customers for a restaurant (Priyadarshini and Cotton 2021). An automatic brand monitoring framework was proposed using Twitter Romanian data. This model effectively generated the reputation report of a single brand, a comparative report of two different companies, and desired time frame (Istrati et al. 2021).

### SA in finance

SA is used to evaluate the financial sector news and helps investors to choose beneficial schemes to invest in. The excessive growth of SA in finance has been seen with the increasing popularity of cryptocurrency. Several cryptocurrencies like Bitcoin, Ethereum, Binance Coin, Quant, Solana, and ZCash are available in the digital finance platform. There is no legislated background available for these cryptocurrencies by which users can faith on them to invest. SA is the only solution that can provide the opinion of different people towards a particular cryptocurrency and helps in decision-making. Machine learning techniques have been used to predict the price movement of Bitcoin, Ethereum, Ripple, and Litecoin cryptocurrencies (Valencia et al. 2019). SA has a wide future scope in cryptocurrency price movement predictions. Researchers are taking a keen interest in this field.

### Technical discussion

SA is widely adopted in different kinds of tasks initiated, from extracting customer opinion (Kumar et al. 2019) toward specific issues to monitoring the patients' mental health based on their posts on social platforms. Additionally, the emergence of new technologies such as Cloud Computing, Big Data (Birjali et al. 2021), Data Science, and Blockchain has widened the field of NLP, including SA. It provided many benefits in the business intelligence domain; companies exploited the SA for customer feedback, product improvement, and marketing strategies (Bernabé-Moreno et al. 2020). SA became a handy tool in cryptocurrency price prediction, Forex prediction, and stock marketing prediction. A recommender system is a model that trains to suggest relevant items (music, movies, or products) to buy. Here, the sentiment analyzer plays a major role in the recommender system for suggesting things (Birjali et al. 2021). SA gathers the opinion of users and feeds the information into the recommender system for final recommendation. Researchers proposed a novel adaptive learning model based on social platform analysis and showed how SA and Big Data could transform e-learning platforms. Furthermore, in government policies and other similar issues, SA is very helpful in monitoring possible public reactions. In the past few years, Twitter has been utilized to analyze the opinion of people toward the global COVID-19 pandemic. SA has been adopted to observe the government strategies (Alaoui et al. 2018), people's reactions, and World Health Organization (WHO) policies as a preventive measure to fight against COVID-19. The Healthcare domain is taking so much interest in SA recently. This allows medical actors to extract information about drug reactions, disease diagnosis, epidemics, and patient moods (Ramírez-Tinoco et al. 2019; Tiwari et al. 2021).

Machine learning is the most promising approach for SA. Usually, machine learning-based SA provides a high accuracy score than the lexicon-based approach.. It offers various feature engineering techniques that extract the critical features from the dataset and improve the efficiency of SA. A graph-based approach connects interrelated words in text reviews to calculate the sentiment and opinion of people where vertices and nodes conform to features available in reviews. Various graph-based methods and algorithms have been applied in the last decades to solve the problem of SA (Tiwari and Kumar 2020; Bhati and Rai 2021). SA improves the classification problem by reducing poor and unfortunate selection. It has the capability and knowledge of various learners, which increases the accuracy of a classification and decreases errors in prediction. The hybrid approach utilizes the capability of various approaches such as rule-based, lexicon-based, machine learning, or deep learning-based. It enhances the efficiency of the SA model with optimum results. It is an idea that generates in a researcher's mind to develop the best approach for a particular task.

The journals "Elsevier" and "Springer" are two more common venues for SA publications. Where Elsevier, Springer, and ACM are three popular publishers that are chosen by researchers for SA-related authentic research. A benchmark dataset plays a vital role in sound research. It has been seen in the graph that researchers more frequently use product reviews for their experiments. Secondly, Twitter gained more popularity among researchers for SA-related experiments. Few researchers also generate their datasets for sentiment classification. Whereas the researchers also consider movie reviews, medical reviews, and hotel reviews for SA-related experiments. SA is an emerging field, but it has various challenges, making it process-critical and decreasing the efficiency of related models. Although researchers are working to solve these issues using discriminant techniques, there is still a lack of accuracy. These challenges generate obstacles to extracting the correct meaning of sentiments and classifying the correct polarity. The number of N features increases the domain dimensionality of the datasets. Feature engineering is a very important step in SA applications and opinion mining. Feature selection and feature extraction should be intractable with final processing in optimal feature engineering. Table 22 summarizes the response of the studies addressing each research question.

**Table 22:** Research findings and questions mapping with references

Research question	Research findings	References
RQ-1	It is discovered that the techniques of sentiment analysis are classified into five main categories, namely the Lexicon-based approach, Machine Learning-based approach, Graph-based approach, Ensemble approach, and Hybrid approach. Where machine learning (SVM) and ensemble learning (Bagging and Boosting) are more popular mechanisms for effective sentiment analysis. As a publishing platforms, Springer, Elsevier, ACM Transaction, and Conference/Workshops are major platforms that are selected by researchers for publishing SA-associated research. Customer reviews on shopping websites are a significant source that is utilized by decision-makers and researchers for the sentiment analysis process	Xia et al. 2011; Taboada et al. 2011; Krishnakumari and Akshaya 2019; Nandi and Agrawal 2016; Sharmaa and Dey 2012; Abdul-Mageed and Diab 2012; Palanisamy et al. 2013; Kundi et al. 2014; Kaushik and Mishra 2014; Abdulla et al. 2014; Bhoir and Kolte 2015; Moreno-Ortiz and Fernández-Cruz 2015; Rajput et al. 2016; Akter and Aziz 2016; Ray and Chakrabarti 2017; Aung and Myo 2017; Alshutayri and Atwell 2017; Khoo and Johnkhan 2018; Dey et al. 2018) Tiwari and Singh 2019; Yerpude et al. 2019; Mowlaei et al. 2020; Atteveldt et al. 2021; Sallam et al. 2022; Rushdi Saleh et al. 2011; User-level sentiment analysis incorporating social networks 2011; Wang and Manning 2012; Habernal et al. 2013; Anjaria and Guddeti 2014; Patil et al. 2014; Le and Nguyen 2015; Wawre and Deshmukh 2016; Dey et al. 2016; Elmurngi and Gherbi 2017; Singh et al. 2017; Hasan et al. 2018) Shi and Li 2011; Jagdale et al. 2019; Kumar et al. 2020; Usman et al. 2021; AlBadani et al. 2022; Aisopos et al. 2011; Ponomareva and Thelwall 2012; Rajagopal et al. 2013; Montejo-Ráez et al. 2014; Castillo et al. 2015; Violos et al. 2016; Chen et al. 2017; Westgate and Valova 2018; Liu et al. 2019; Bordoloi and Biswas 2020; Chen et al. 2021; Liang et al. 2022) Ying et al. 2012; Sharmab and Dey 2013; Fersini et al. 2014; Wang et al. 2014; Chalothom and Ellman 2015; Perikos and Hatzilygeroudis 2016; Lochter et al. 2016; Onana et al. 2016; Onanb et al. 2016; Heredia et al. 2017; Prashanth. Avverahalli Ramesha. 2017; Akhtar et al. 2017; Saleena 2018; Bari and Saatcioglu 2018; Kim and Park 2019; Khan et al. 2019; Khalid et al. 2020) Nazeer et al. 2021; Bibi et al. 2022; Rahman and Islam 2022; Villena-Román et al. 2011; Sohn et al. 2012; Govindarajana 2013; Revathy and Sathiyabhama 2013; Govindarajanb 2014; Al-Twairesh et al. 2014; Jasmine Bhaskar and Sruthi, and Prema Nedungadi. 2015; Asghar et al. 2016; Mondal et al. 2016; Keith et al. 2017; Tripathy et al. 2017; Amrani et al. 2018; Zainuddin et al. 2018; Srivastava et al. 2019; Joshi and Gupta 2019; Elshakankery and Ahmed 2019; Chaithra 2019; Benlahbib and Nfaoui 2020; Jing et al. 2021; Tiwari and Nagpal 2022)
RQ-2	With the increasing demand for SA, several challenges has also enhanced, such as stance detection, sarcasm detection, negation-handling, domain dependence, huge lexicon, word-sense disambiguation, and anaphora resolution. A stance dataset is created, and a neural model has employed for detecting the favorability of the text toward a given target. The "Sentence-Related" features, "Punctuation-Related" features, "Syntactic and Semantic" features, and "Pattern-Related" features are proposed by researchers to handle sarcasm in text. Linguistic feature-based and LSTM-linguistics have been utilized for negation handling. The SFA algorithm and BERT have been trained to reduce the challenge of domain dependence. Various lexicon dictionaries are initiated by researchers to identify the meaning of words and solving the problem of word-sense disambiguation	Mohammad 2016; Augenstein et al. 2016; Sun et al. 2018; Cabral and Hortacsu 2010; Bouazizi and Ohtsuki 2016; Joshi et al. 2015; Farooq et al. 2017; Gautam et al. 2018; Pan et al. 2010; Peng et al. 2018; Chunning et al. 2020; Hong and Hatzivassiloglou 2003; Minqing and Liu 2004; Ge et al. 1998; Lappin and Leass 1994)
RQ-3	The number of N features increases the domain dimensionality of the datasets. Feature engineering is a very important step in SA applications and opinion mining. Feature selection and feature extraction should be intractable with final processing in optimal feature engineering. Dimensionality reduction reduces the high dimensions of the dataset, which keeps more discriminative and constructive features from the collection set. Feature engineering is categorized into two major parts (1) Feature Extraction and (2) Feature Selection. Feature extraction is a process of selecting required or essential features from the original set. Principal Component Analysis (PCA) and Latent Semantic Analysis (LSA) are the two popular techniques of feature extraction. At the same time, feature selection is a process that reduces the number of variables for predictive models. Effective and efficient feature selection improves the performance of SA. The feature selection process includes missing values removal, low variance removal, highly correlated feature removal, univariate selection, and recursive elimination. Feature selection methods are categorized into two groups: filter methods and wrapper methods that are utilized for effective SA process	Kohavi and John 1997; Liu et al. 2020; Zareapoor and Seeja 2015; Uysala and Gunal 2014; Gomez et al. 2012), Kumar et al. 2017; Dasgupta et al. 2007; Guyon and Elisseeff 2003; McHugh 2013; Sharmac and Dey 2012; Yang and Pedersen 1997; Alper Kursat Uysalab 2016; Uysalc and Gunal 2012; Baccianella et al. 2013; Inza et al. 2004; Alper Kursat Uysald 2018; Mousin et al. 2016; Lei 2012; Sharkawy et al. 2011)
RQ-4	Emotion extraction and classification are essential parts of SA. So, here in this survey, the major emotions are concluded that are considered by the various researchers in SA during facial expression, gesture presentation, motion, and voice recognition. Here, a standard emotion set has been that is common in various types of research. Automatic human facial expression extraction is an emerging application of Human–Computer Interaction (HCI) and affective computing. Therefore, emotion extraction and classification became prime aspects in the research field of SA. Several researchers have been working on a distinctive set of emotions and expressions	Gunesa and Piccardi 2005; Gunesb and Piccardi 2008; Hablani et al. 2013; Chen et al. 2013; Hayat and Bennamoun 2014)
RQ-5	The ensemble approach of machine learning has been used in various applications and has produced outstanding results. Ensemble learning is also approachable in the SA task. Therefore, we have presented a comparative analysis of diverse ensemble methods that are divided into two main categories: bagging and boosting. This study compares eight popular ensemble learners (Random-Forest, Extra-Tree, Meta-Estimator (Linear SVC), Ada-Boost, Gradient-Boosting, XGB, Cat-Boost, and Light-GBM) to choose the best model for SA. The experiments have been conducted on four different domain reviews: Uber reviews, Restaurant reviews, Amazon reviews, and Food reviews. After analyzing the comparative results presented in Sect. 5, It is stated that bagging-based ensemble techniques (Random-Forest, Extra-Tree, and Meta-Estimator (Linear SVC)) performed better than boosting-based techniques. Random-Forest and Extra-Tree perform almost equally. Meta-Estimator (Linear SVC) gives less training accuracy and testing accuracy than Extra-Tree and Random-Forest but provides higher speed comparatively. However, XGB and Cat-Boost obtain better accuracy and TPR than other boosting ensembles but cannot beat the performance of bagging-based ensembles. Hence, bagging ensemble-based techniques provide state-of-the-art results for SA	Frequency xxxx; Polikar 2006; Random-Forest. xxxx; Geurts et al. 2006; Sharaff and Gupta 2019; Freund and Schapire 1996; Drucker et al. 1994; Torelli and Menardi 2008; Bahad and Saxena 2020; Friedman 2002; Meng et al. 2016; Nguyen et al. 2018; Bhati et al. 2020; Zhang et al. 2019; Bichitrananda Behera and Kumaravelan, and Prem Kumar. 2019; Abualigah et al. 2020; Barkur and Vibha 2020; Basiri et al. 2021; Denecke and Deng 2015; Ramteke et al. 2016; Neogi et al. 2021; Somula et al. 2016; Priyadarshini and Cotton 2021; Istrati et al. 2021; Valencia et al. 2019; Kumar et al. 2019; Birjali et al. 2021; Bernabé-Moreno et al. 2020; Alaoui et al. 2018; Ramírez-Tinoco et al. 2019)

## Conclusions and future work

This article presents an immense literature survey of 92 reputed articles, which includes lexicon-based, graph-based, machine learning-based, ensemble-based, and hybrid-based techniques for SA. It is observed that ensemble-based and hybrid-based techniques gained more popularity for text classification. In addition, essential aspects such as frequently used SA datasets, publishing platforms, proposed techniques, SA challenges, SA feature-engineering techniques, and various emotion theories are also discussed in this study. With the rapid demand for SA, several challenges are also occurred in processing the text reviews. So, we discussed several SA-related challenges, namely stance-detection, sarcasm-detection, negation-handling, domain-dependence, huge-lexicon, word sense disambiguation, and anaphora resolution, with their proposed solutions. The feature-engineering is a prime factor for effective text classification. Here we convey an extensive taxonomy of feature-engineering techniques used for text processing. The emotion theory of five admired researchers has also been discussed. The essence of their idea represents disgust, fear, anger, happiness, and sadness, mainly included in basic emotion classification from the text information.

Our primary objective is to provide great relevance to the companies for selecting a better sentiment model for their brand monitoring and product reviews. This article also implemented numerous ensemble-based techniques on different domain reviews datasets, providing a systematic comparative analysis of bagging and boosting-based ensemble for SA. We have also illustrated the core of ensemble-based techniques for SA. Five boosting-based ensembles and three bagging-based ensemble techniques have been implemented on four text review datasets to conduct extensive experiments. The previously discussed ensemble-based research, incorporated with experimented results, provides a broad perspective to apply ensemble-based techniques for SA. Finally, experimental results demonstrate that bagging-based ensemble techniques outperform in terms of TPR, FPR, accuracy, weighted precision, weighted recall, weighted f1-score, AUC-score, and run-time for SA. However, XGB and Cat-Boost from boosting approach produced effective results but were unable to beat the performance of bagging-based ensembles. This survey with an analytical study will help in determining the best technique for preparing SA-related applications. For future contributions, we will explore the hybrid approaches, where discriminant techniques and models are combined to develop a better model for SA with reduced computational cost. The goal is to develop a hybrid model for SA application with a combination of different approaches. Therefore, we will assess the effectiveness and reliability of the hybrid methods with different types of parameters.

## Data Availability

Uber-Reviews: https://www.kaggle.com/code/hershyandrew/uber-reviews-text-analysis/data. Food-Reviews: https://www.kaggle.com/datasets/snap/amazon-fine-food-reviews. Amazon Reviews: https://www.kaggle.com/code/saurav9786/recommender-system-using-amazon-reviews/data. Restaurant Reviews: https://www.kaggle.com/datasets/d4rklucif3r/restaurant-reviews
